# Predicting Single-Stranded DNA Oligonucleotides 3D Structures: An Open Issue

**DOI:** 10.34133/csbj.0127

**Published:** 2026-06-08

**Authors:** Selma Bengaouer, Thomas Binet, Stéphane Octave, Séverine Padiolleau-Lefèvre, Bérangère Avalle, Irene Maffucci

**Affiliations:** Université de technologie de Compiègne, CNRS, UPJV, GEC, Compiègne, France.

## Abstract

Single-stranded DNAs (ssDNAs) play major biological functions and represent an interesting biotechnological tool. They constitute a compelling alternative to RNA, because of their greater stability as compared to the latter. As for RNA, ssDNAs function depends on the specific foldings they adopt. Therefore, information about ssDNAs 3-dimensional (3D) structures is fundamental to investigate their functions. In this context, *in silico* 3D structure prediction can facilitate ssDNA design. This task can be addressed indirectly, by using the tools for RNA structure prediction and then converting the output in the ssDNA format, or one of the few tools capable of directly handling ssDNA. This study assessed 3 indirect RNA 3D structure prediction methods (RNAComposer, SimRNA, and Vfold3D), based on their performance in the Critical Assessment of Structure Prediction and one direct DNA prediction tool (3dDNA) to evaluate their performances in modeling ssDNAs. At this scope, a dataset of 97 experimentally determined ssDNA structures, including challenging motifs such as G-quadruplex, was built. Various metrics, namely, Root Mean Square Deviation, Global Distance Test Total Score, and Interaction Network Fidelity, were employed to benchmark the accuracy of the predictions. The 3 indirect tools showed similar and moderate performances, while the direct tool provided better results. Nevertheless, they all performed poorly in modeling G-quadruplexes and structures containing motifs increasing the intrinsic flexibility of ssDNA. Despite the recent efforts in the prediction of the 3D folding of ssDNAs, improvements in method are still needed. This should involve taking into account the conformational variability of this kind of molecules and paying attention to their specific 3D motifs.

## Introduction

Single-stranded DNA (ssDNA) oligonucleotides are of major importance in many biological processes [1], and they also represent a powerful biotechnological tool: they can be used as genome editing and engineering elements [2,3], as biosensors [4], and as diagnostic and therapeutic molecules in the form of aptamers [5–7]. Moreover, there is a growing interest toward ssDNA [8]. This is due to their well-known synthesis routine [9] and greater stability as compared to RNA, resulting from the absence of the 2′-hydroxyl group found in RNA, which makes DNA less prone to hydrolysis compared to RNA [10].

**Fig. 1.**
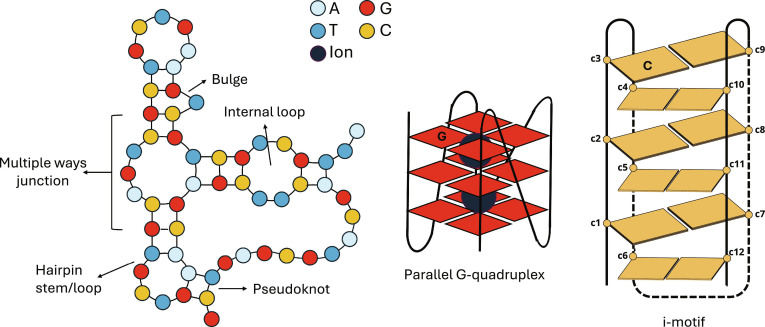
Main motifs found in single-stranded DNA.

ssDNAs, and single-stranded oligonucleotides (ssNAs) in general, are highly flexible and, thanks to intramolecular base pairing, they can fold into specific 3-dimensional (3D) conformations, which are crucial for their stability and function. Because of their intrinsic flexibility, ssNA structures are characterized by a large variety of base pair networks, named secondary structure motifs, such as hairpins, bulges, internal loops, G-quadruplexes, pseudoknots, and multiple-way junctions (Fig. 1). These form the basis for higher-order 3D architectures, where additional interactions, including long-distance contacts and molecular stacking, further refine the stability and functionality of ssNAs [11]. This structural richness enables them to bind a wide range of target molecules with remarkable specificity and affinity, achieving dissociation constants in the nano- to picomolar range [6,7,12].

According to this view, a high-resolution structural determination is crucial to understand the biophysical properties and functional mechanisms of ssNAs, including ssDNAs. However, the characterization of their 3D structures at an atomic resolution remains a fundamental challenge. Indeed, experimental techniques exploitable at this scope are often complex, expensive, and time-consuming [5,12–14]. Computational methods for ssNA 3D structure prediction provide a promising alternative, generating valuable insights that complement experimental findings. As a consequence, similarly to the protein folding problem [15], the 3D structure prediction of ssNAs is becoming a bioinformatics hot topic, to the point that this kind of molecules have been included in the latest Critical Assessment of Structure Prediction (CASP) challenges [16,17]. In this context, more than 40 methods for the prediction of RNA 3D structures were assessed on both RNA and a few ssDNA target sequences, though the overall results show that the performances of these algorithms are still very far from those of the methods for the 3D structure prediction of proteins.

It has to be noted that the prediction performances in modeling ssDNA were poorly assessed: first of all, none of the few available tools dedicated to this kind of ssNAs was tested. Furthermore, although the procedure of using an RNA structure prediction tool followed by a structural conversion and minimization to model ssDNA is well known [18], referred from now on as the indirect procedure, no ssDNA was present in the CASP15 blind challenge and only 2 ssDNA were included in the CASP16, with one being in complex with a protein [17].

Besides the indirect RNA-based approaches, some tools have been developed to directly model the structure of DNA [19] (direct prediction tools) and have been tested outside the CASP challenges. However, these methods are often focused on double-stranded DNA or they require a preliminary generation of a topological and an initial configuration file, hampering a straightforward usage or potentially biasing the prediction depending on the quality of the provided initial coordinates.

Therefore, in order to thoroughly and consistently define the state of the art for ssDNA structure prediction, we selected 4 ssNA structure prediction tools freely available as servers or stand-alone programs, namely, RNAComposer [20], SimRNA [21], Vfold3D [22], and 3dDNA [23,24]. The first 3 ranked among the top 5 in the CASP15 and/or CASP16 challenges and have been developed for RNA structure prediction. Conversely, 3dDNA was designed to directly predict dsDNA and ssDNA structures and, in contrast to other similar available tools [25,26], it does not need any preliminary information about the topology and configuration of the ssDNA. The assessment of the ability of these tools in reproducing the experimental structure was evaluated on 97 ssDNAs retrieved from the Protein Data Bank (PDB) [27] and the Nucleic Acid Database [28]. The built dataset included a variety of motifs and ssDNA both in the free state and in complex with proteins, allowing for a comprehensive assessment of structural predictions under different biological contexts.

The predictive accuracy of the aforementioned methods was evaluated using multiple structural metrics. Overall structural similarity was evaluated using 2 measures: the heavy-atom Root Mean Square Deviation (RMSD) and the Global Distance Test Total Score (GDT_TS) [29], while the accuracy of predicted molecular interactions was assessed with the total Interaction Network Fidelity (INF) [30].

**Fig. 2.**
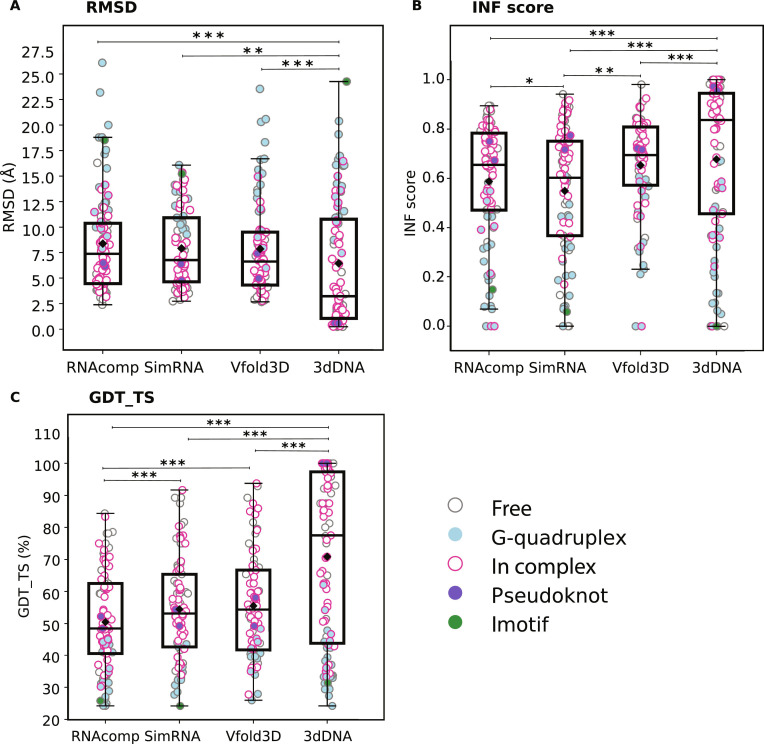
Boxplots of (A) heavy-atom RMSD, (B) INF score, and (C) GDT_TS obtained for the models provided by RNAComposer, SimRNA, Vfold3D, and 3dDNA for the 97 ssDNA structures. The mean values are indicated as black diamonds; the G-quadruplexes, the pseudoknots, and i-motif are indicated as light blue, purple, and green dots, respectively, while the contour of the dots indicates the ssDNA state: free (gray) and in complex with a protein (magenta). *P* values < 0.001 (***), <0.01 (**), and < 0.05 (*) resulting from the application of the Wilcoxon test for paired samples are marked. Full details of *P* values are reported in Table S5.

This enabled a comprehensive evaluation of how accurately RNA/DNA structure prediction tools can model ssDNA structures, highlighting both their strengths and limitations. 3dDNA, the structure prediction tool designed for directly modeling ssDNA, overall provided the best performances in producing models close to the experimental structures, all metrics considered (Fig. 2). Nevertheless, this tool is also the one with the widest value distributions for the 3 metrics (Fig. S1), suggesting that it either provides excellent models or fails in the task. In addition, all considered tools encountered difficulties in modeling ssDNA containing G-quadruplexes (G4) or with a particularly high conformational freedom, given by loose ends and multiple loops. These results indicate that much effort has still to be done to generate reliable ssDNA structures characterized by the presence of canonical and noncanonical base pairs. Additionally, special attention should be paid to explore the ssDNA conformational space.

## Materials and Methods

### ssDNA dataset

The dataset used in this study consists of ssDNA sequences having a published experimental 3D structure retrievable from the PDB [27] and the Nucleic Acid Database [28]. All the structures containing a DNA were initially retrieved, then only those containing ssDNA were conserved. Additionally, sequences containing non-natural nucleotides or corresponding to hybrid DNA and RNA sequences were discarded, together with structures with missing nucleotides and those in complex with small molecules. Indeed, in this latter case, usually the ssNA folds around the target molecule, ending up in a conformation very far from the free state one. Finally, redundant sequences were filtered, in order to have a well-diversified dataset in terms of motifs and sequences. The resulting dataset comprises 97 ssDNAs (Table S1), ranging from 7 to 53 nucleotides and including 36 simple hairpins, with a number of base pairs ranging from 2 to 15, 2- to 5-nucleotide-long hairpin loops, and 0 to 10 unpaired nucleotides outside the hairpin. Moreover, the dataset comprises 19 hairpins containing bulges and/or loops (either symmetrical or asymmetrical), 4 multiple-way junctions, 7 structures with one isolated base pair, 2 oligonucleotides with a long-distance interaction similar to a pseudoknot, 1 oligonucleotide adopting an i-motif conformation, a triple helix, a structure composed by 2 independent hairpins, and 26 oligonucleotides possessing a G4 motif (with or without other motifs). Among these, 12, 9, and 4 structures contain parallel, antiparallel, and hybrid G4, respectively. This ensemble of structures ensures the variability of the dataset and limits the possible prediction biases.

For each ssDNA, the experimental secondary structure was extracted from its 3D structure using x3DNA-DSSR [31]. In the case of ssDNAs containing G4 motifs, a hybrid approach was applied: the G4 secondary structure was first predicted using ElTetrado [32], then integrated with the secondary structure obtained via x3DNA-DSSR to reconstruct the complete and accurate 2-dimensional (2D) structure. The secondary structure of the i-motif oligonucleotide was generated manually, since none of the available automatic tool can handle this peculiar secondary structures.

### 3D structure prediction tools

For the designed benchmark, we chose to test the predictive capabilities of 4 ssNA 3D structure prediction tools freely available as stand-alone programs for Unix/Linux distributions or as free web servers. Three of them, namely, Vfold3D [22], SimRNA [21], and RNAComposer [20], although mainly developed for RNA, were chosen because of their performances in CASP15 and/or CASP16 challenges [16,17]. The fourth, 3dDNA [23,24], is specifically suited for the prediction of DNA structures and showed good performances in other studies [24].

Vfold is a computational method for predicting the secondary and/or 3D structure of RNA molecules. The software consists of 2 algorithms, Vfold2D and Vfold3D. However, in this study, unless otherwise stated, we focused on Vfold3D, since the experimental secondary structure of each ssDNA was provided as input together with its sequence. The Vfold3D algorithm uses the motif template assembly method [22] to predict the target 3D structure. The process begins with the identification of structural motifs in the target RNA based on parameters such as sequence length and similarity. These motifs are then searched within the Vfold3D motif template database, which has been built from RNA structures available in the PDB. If specific motifs are absent or not represented in the database, Vfold3D may not accurately predict the target structure. In such cases, the VfoldLA algorithm [33] is employed. It uses a similar assembly strategy, but is based on loop templates and helices instead of motif templates to predict unidentified motifs. The assembled structures may exhibit structural clashes or non-ideal features, such as incorrect bond lengths, bond angles, or torsion angles. To efficiently optimize these structures without excessive computational cost, the algorithm uses the IsRNA model [34]. This model combines coarse-grained Replica Exchange Molecular Dynamics (REMD) simulations with knowledge-based interaction potentials to refine the all-atom structures generated during the assembly. Therefore, in our study, for each initial 3D structure of our database, REMD simulations were performed using 10 replicas. The simulations were run with default parameters, including a simulation time of 5 ns and 1,000 recorded structures per replica. The algorithm generated 5 final predicted structures based on the clustering of the low-energy structures of the trajectories.

SimRNA is a stochastic computational method for predicting the 3D structure of RNA molecules. It is built on 3 main functional components: (a) the representation of the simulated molecule by using a coarse-grained model, (b) a scoring function used to guide the conformational search, and (c) a method for the sampling of the conformational space. According to the SimRNA coarse-grained model, every nucleotide is represented by 5 beads: 2 beads describe the backbone, namely, 1 for the phosphate group and another 1 for the sugar moiety, while the bases are represented by 3 beads. The scoring function guiding the conformational search assigns a numerical score to each RNA conformation by calculating various terms that capture the physical and chemical properties of RNA (e.g., the base pairing interactions, the base stacking interactions, the torsion angles, and the distance restraints). Finally, the sampling method employed by SimRNA makes use of the Monte Carlo sampling in either single-thread simulations or replica exchange simulations. In this study, the parameters recommended by the developers have been used. The bonds, angles, and torsion angle weights that impact the energy of the system are reported in Table S2.

RNAComposer is an algorithm implemented on a web server that can predict the complete 3D structure of RNA molecules. The method relies on assembling short fragments extracted from known structural data, which are then combined to reconstruct the full structure. The algorithm uses the RNA FRABASE [35,36] database to identify and locate the building blocks corresponding to the previously identified short fragments. To use this algorithm, the default parameters were employed. Notably, 800 structures are initially generated and minimized through 20,000 Monte Carlo cycles. The resulting structures are then ranked by energy and the top 10 are conserved.

3dDNA [23,24] is a template-based method capable of predicting the 3D structure of a target DNA from its sequence and secondary structure. The process begins with the decomposition of the target DNA secondary structure into elements (SSEs). These SSEs are used to find a suitable corresponding 3D structure initially in the 3dDNA_lib (the DNA SSE 3D templates), and, in case of failure, in the 3dRNA_lib (the RNA SSE 3D template library). If the algorithm fails to find a 3D template in both libraries, a distance geometry and a bi-residue algorithm are used to build a 3D template for this SSE. Successively, the algorithm moves to the assembling step guided by the 2D structure. This is followed by a short minimization to avoid any artifacts. If 3D templates are available for all SSEs in the 3dDNA_lib, the assembled structure is returned as the final model of the target DNA. If it is not the case, this structure is subsequently optimized using the residue-level simulated annealing Monte Carlo (SAMC) method, guided by the modified residue-level energy function implemented in 3dRNA by replacing U with T. The same scoring function is used to rank the optimized structures, and the top 5 models are provided as output. In this study, we used the web server version of the method, which offers 3 prediction modes: (a) assemble, in which the algorithm performs only the assembly step and, if necessary, applies an additional optimization using SAMC method followed by energy minimization; (b) optimize, in which the algorithm systematically performs both the assembly and optimization steps; and (c) default, in which the algorithm automatically determines whether to return the assembled model or to apply the optimization step, selecting the best-performing model between the two. Based on these considerations, the entire dataset was evaluated using the different prediction modes. For each structure, we excluded, during the computation, all the PDB structures associated to the ssDNA to be modeled. The assemble mode yields the most accurate prediction models and is therefore used for the remainder of this study.

For the 4 selected tools, the experimental secondary structure has been provided as input in order to evaluate the predictions quality starting from a unique and experimentally correct base pair network.

### Conversion from RNA to DNA after indirect tool predictions

RNAComposer, SimRNA, and Vfold3D, as all the indirect prediction tools, are designed to predict RNA structures on the basis of a RNA input sequence. Vfold3D and RNAComposer can process DNA sequences by automatically converting them into RNA structures before proceeding with the prediction step, while SimRNA requires as input an RNA sequence. In all cases, the output is in RNA format. Therefore, the obtained RNA structures were manually converted into ssDNA structures by mutating the uracil bases into thymine bases using the PyMOL mutagenesis plug-in and replacing the hydroxyl group in C2′ position by a hydrogen atom.

Successively, a structural minimization was performed in the gas phase using the Amber20 package [37]. This involved the minimization of the hydrogens through 4,000 steepest descent cycles followed by up to 1,000 conjugate gradient cycles. The whole structure was then successively minimized over 16,000 steepest descent cycles and up to 4,000 conjugate gradient cycles.

### Comparison 3D structure metrics

To assess the efficiency and accuracy of the selected tools, each predicted ssDNA structure was compared to the corresponding experimental reference structure. Various metrics were used to evaluate both global and local accuracy, in agreement with the guidelines of the CASP15 and CASP16 international challenges [16,17].

In the light of this, we computed the RMSD as

$$\text{RMSD}=\sqrt{\frac{1}{N}\sum_{i=1}^{N}\delta_{i}^{2}}$$ (1)

where $\delta_{i}$ corresponds to the distance of the atom *i* of the predicted structure from the same atom in the reference one. In our case, we considered the RMSD of the heavy atoms of the predicted structure from the same set of atoms of the experimental structure. In accordance with the literature [38,39], we considered that a prediction was close to the reference structure when the RMSD was lower than 5 Å.

The GDT_TS was calculated using the Local Global Alignment tool [29]. This metric evaluates the percentage of C4′ atoms in the predicted structure that fall within predefined distance thresholds of 1, 2, 4, and 8 Å. These thresholds indicate the level of structural similarity between the predicted and reference structures. The GDT_TS is derived by summing the percentages calculated for each threshold, and it ranges from 0% (no similarity) to 100% (perfect match) [Eq. (2)]. We considered that the prediction globally reproduces the experimental folding when the GDT_TS is greater than 45% [40].

$$\begin{array}{c} \mathrm{GDT}=\\ \frac{1}{4}\sum_{D\in \left\{\;1,\;2,\;4,\;8\;\right\}}\left(\frac{\text{Number of aligned residues within dist}\leq d}{\text{Total number of residues}}\right) \end{array}$$ (2)

The total INF score [30] was incorporated in this study to evaluate how well a predicted molecular structure preserves the network of interactions present in a reference structure. The total INF score is computed as:

$$\mathrm{INF}=\sqrt{\left(\frac{\mathrm{TP}}{\mathrm{TP}+\mathrm{FP}}\right)\left(\frac{\mathrm{TP}}{\mathrm{TP}+\mathrm{FN}}\right)}$$ (3)

where TP (true positive) is the number of interactions (the canonical and noncanonical base pair and the base-stacking, in this study) correctly predicted in the model that correspond to those in the reference structure. FP (false positives) is the number of interactions predicted in the model that do not have a corresponding match in the reference structure. FN (false negative) is the number of interactions present in the reference structure but missing from the predicted model. The INF score ranges from 0 (low fidelity, the reconstructed network does not accurately reflect known interactions) to 1 (high fidelity, the reconstructed network captures a large proportion of the interactions present in the reference network), with values ≥0.7 indicating a globally satisfying prediction of the interaction network [41].

In order to verify the statistical significance of the differences obtained for each considered metrics by each tool, the bilateral Wilcoxon test for paired samples was performed.

### Secondary structure comparison metrics

As a further analysis, the secondary structure of each predicted model was compared to the experimental one. This required, as a first step, the conversion of each modeled 3D structure into its corresponding 2D representation in the dotbracket notation using the stand-alone version of x3dna- dssr [31]. As previously mentioned, for the structures containing G4 motifs, a combined strategy using both x3dna- dssr [31] and Eltetrado [32] was used. The resulting 2D structure was then compared to the experimental 2D structures initially provided to the algorithms.

The comparison was made using AptaMat [42] as metric, a highly sensitive matrix-based algorithm designed specifically for comparing aligned secondary structures of single-stranded nucleic acids. An AptaMat score of 0 indicates that the 2 compared secondary structures are identical, an AptaMat score ≤1.5 refers to close secondary structures, and an AptaMat score > 1.5 suggests that the 2 compared secondary structures differ [43]. When the AptaMat score could not be computed, due to the absence of base pairs in 1 of the 2 compared secondary structures, a value of −1 has been indicated.

## Results and Discussion

To define the state of the art for ssDNA 3D structure prediction, we assessed the performance in reproducing the experimental structure of a target ssDNA of 4 different tools: RNAComposer [20], SimRNA [21], Vfold3D [44], and 3dDNA [23,24]. These have been selected among all the published tools on the basis of multiple criteria. First of all, for each of them, either a stand-alone version or a web server implementation is freely available. In addition to this, RNAComposer, SimRNA, and Vfold3D were selected based on their strong performance in the CASP15 and/or CASP16 challenges. Although they were originally developed for RNA 3D structure prediction, in the latest CASP (CASP16), they were also applied to ssDNA, though on only a few structures. Indeed, the indirect approach, consisting of using an RNA structure prediction tool and then converting the output to ssDNA, is well known and has already provided acceptable results [18]. Conversely, 3dDNA was chosen because it takes inspiration from a RNA 3D structure prediction tool (3dRNA [45]), but it has been developed for DNA, either single- or double-stranded. As described in Materials and Methods, it employs a specific DNA secondary structure elements library for building the DNA model, and only in case of absence of correspondences does it make use of an RNA motif library. Because of this, 3dDNA is an ideal tool to investigate whether including DNA-specific terms or elements during the prediction is significantly more effective in terms of structural accuracy compared to RNA-based approaches adapted to DNA. In addition, as compared to other ssDNA structure prediction tools [19,25,26], it has been specifically developed for structure prediction and does not require any initial configuration or topology. Indeed, the latter could positively or negatively bias the prediction.

The prediction performances of these tools were tested on a dataset of 97 experimentally determined ssDNA structures with sequence lengths ranging from 7 to 53 nucleotides (Table S1 and Supplementary Materials). The dataset comprises all kinds of structural motifs (Fig. 1), including G4, pseudoknots, and even an i-motif (see Materials and Methods for a detailed description of the dataset content). This allows to extensively challenge the chosen tools, together with the inclusion of 49 ssDNAs whose structure was resolved in complex with a protein. Indeed, ssNAs are characterized by a set of conformations in dynamical equilibrium, which can be shifted and perturbed by interaction with another molecule [46]. It is therefore interesting to investigate how the selected tools behave in this scenario.

As previously mentioned, RNAComposer, SimRNA, and Vfold3D are developed to model RNA structures. Therefore, for these tools, an RNA-to-DNA conversion is required before assessing the quality of the modeled structures (see Materials and Methods for details). Briefly, the used conversion approach consists of a manual mutation of the sugar and, eventually, the nitrogenous base, followed by a gas-phase minimization, and is coherent with what was reported in the literature [18]. Although the structural difference between RNA and ssDNA is well known [47], this procedure allows testing the performances of the indirect tools in predicting ssDNA structures, avoiding biases coming from additional steps following the prediction, where explicit water molecules and ions might be included and would affect the prediction.

In this context, it is worth mentioning that none of the selected tools allows to explicitly include ions or solvent during the prediction. However, the effect of the environmental conditions, known to play a significant role for oligonucleotides structures, is indirectly taken into account by the different tools, either within the fragment libraries used for the structure reconstruction (e.g., Vfold3D) or by the potentials of the SimRNA coarse-grained model.

Successively, independently from the prediction tool, each generated model was compared to the corresponding experimental structure by using different metrics commonly used to collate oligonucleotide 3D structures, namely, the heavy-atom RMSD, the total INF score, and the GDT_TS, which can provide additional information about the quality of their topologies (Table S3). In addition to the analysis of the scores’ distributions and statistics, to facilitate the analysis and discussion of the results, for each metric, a quality threshold was fixed according to the literature. More precisely, a good quality model should have a heavy-atom RMSD ≤ 5 Å from the experimental structure [3,47], an INF score ≥ 0.7 [40], and a GDT_TS ≥ 45% [27]. Although these thresholds have been mainly applied to RNA structures, their applicability to ssDNA was verified by a visual inspection of the obtained ssDNA models. In addition, the global coherence of the results obtained by the 3 different metrics, each of them capturing different elements of the structures comparison, supports the threshold choice.

### Global analysis of prediction tool performances

Globally inspecting the provided predictions, it has to be noted that RNAComposer and SimRNA were able to successfully generate 3D models for the whole ssDNA dataset, while Vfold3D and 3dDNA failed to produce 16 and 2 models out of 97 structures, respectively (Table S4 and Supplementary Materials). The 16 structures not predicted by Vfold3D include 7 ssDNAs containing a G4 motif, 8 very short sequences (ranging between 7 and 14 nucleotides), and 1 structure containing an i-motif. Except for the i-motif, where no canonical base pair was observed, these structures have a common characteristic: their secondary structure is characterized by a single isolated canonical base pair, which makes the folding prediction particularly challenging. In contrast, the 2 structures not predicted by 3dDNA (1KR8 and 1PQT) correspond to very short sequences, both consisting of 7 nucleotides. As a consequence, the overall performance of the Vfold3D and 3dDNA tools are slightly incorrectly estimated due to their failure to predict 16.49% and 2.06% of the dataset, respectively.

In addition to this, the 3 indirect prediction tools provide medium quality and overall similar results [*P* values > 0.05 (Table S5), and between 30% and 40% of predictions with RMSD ≥ 5 Å], with none of them excelling in predicting the ssDNAs’ 3D structures and, at the same time, completely failing in the task (Fig. 2). They show relatively similar median heavy-atom RMSD values: 7.4, 6.8, and 6.6 Å for RNAComposer, SimRNA, and Vfold3D, respectively, which are all above the threshold of 5 Å (Fig. 2A and Table S7). Moreover, it is relevant to mention that these indirect tools succeed in providing a good quality model most of all for ssDNA mainly characterized by the presence of simple hairpins. This suggests that, independently from the structural differences between RNA and ssDNA, indirect tools are potentially capable of dealing with the ssDNA structural preferences when simple motifs are involved.

Conversely, 3dDNA prediction performances are significantly better than those of the indirect tools (*P* values < 0.01, Table S5 and Fig. 2). Indeed, it provided a median heavy-atom RMSD of 3.23 Å, with 56.8%—calculated over the 95 structures 3dDNA handled—of the structures with an RMSD below 5 Å (Table S7). This indicates that 3dDNA is effective in generating accurate structural models, when it is able to provide a prediction for a given sequence and secondary structure. However, Fig. 2 and Fig. S1 clearly show a wider distribution of the RMSD values of the 3dDNA models as compared to those produced by the tested indirect tools, and an average RMSD value (6.45 Å, Table S7) almost doubling the median value. Furthermore, in contrast to what is observed for the indirect prediction tools, the 3dDNA RMSD distribution is almost bimodal, suggesting that this tool either provides a model of excellent quality (RMSD < 2 Å) or completely fails in the task, with models showing a heavy-atom RMSD > 10 Å, and just a few in the 5- to 10-Å RMSD range. Conversely, SimRNA is the tool providing the narrowest RMSD distribution for its models, with no model having an RMSD above 17.5 Å (Fig. 2A). The origin of the peculiar 3dDNA behavior potentially comes from the pipeline followed by 3dDNA (see Materials and Methods). Indeed, if the smallest secondary elements (SSEs) composing an ssDNA are found in the DNA library, the provided model will be excellent. Conversely, if DNA SSEs are not found, a search in the RNA library is performed or additional algorithms are applied for the generation of the model. This alternative procedure will fall back to a semi-indirect modeling approach, which might provide models of low quality. This can represent a decision element when wanting to generate an initial ssDNA configuration for further analysis, such as dynamics or interaction studies.

The RMSD has notable limitations, such as its dependence on the molecule size, its sensitivity to the structure alignment, and its difficulty in capturing local details, highlighting the need for additional complementary metrics. Therefore, in order to avoid interpretation biases due to the threshold choice and to confirm the global analysis, we selected 2 additional metrics: the GDT_TS, and the total INF score. The latter allows the investigation of the quality of the interaction network (all kind of base pair and base stacking) of the model as compared to the reference structure.

Figure 2B shows the results obtained for this metric by the 4 prediction tools. Coherently with the observations made when analyzing the RMSD, a strongly significant difference is observed between 3dDNA and the indirect tools (*P* values < 0.001, Table S5): 3dDNA achieves the best median INF score (0.84). Conversely, Vfold3D, RNAComposer, and SimRNA modeling resulted in a median INF score of 0.69, 0.65, and 0.61, respectively, and a weaker significant difference only between RNAcomposer and SimRNA (*P* value < 0.05), and between SimRNA and Vfold3D (*P* value < 0.01) (Table S5). When focusing on the models characterized by a high INF score, 3dDNA provided 57.8% (over 95 structures) of the models above the fixed threshold, while this percentage is of 56% (over 81 structures) for Vfold3D, and 50% for RNAComposer and SimRNA. This can be translated to a similar performance in correctly reproducing the ssDNA interaction network for the 4 considered tools, with 3dDNA being slightly more performing as compared to the indirect prediction tools.

The GDT_TS score is used to assess the global similarity between a model and a reference structure. In this case, all the observed differences are significant (*P* values < 0.001, Table S5), with the exception of that between SimRNA and Vfold3D. *Vis-à-vis* this metric, RNAComposer exhibits the worst performance, with a median GDT_TS of 48.4% (Fig. 2C) and 56% of the predictions above the threshold of 45%. This, together with the previous results, indicates that RNAComposer can provide models with interactions quite close to those of the reference structure, but suffers from limits in providing a correct spatial organization. Conversely, SimRNA and Vfold3D display better and comparable performances, with median GDT_TS values of 53.1% and 54.4%, respectively, and 64% of their predicted models exceeding the fixed threshold. As previously observed for RMSD and INF scores, the 3dDNA tool exhibits the best GDT_TS median (77.5%) and outperforms the other tools with 71.1% of predicted structures with a score above the fixed threshold. Nevertheless, independently from the considered metrics, 3dDNA showed the widest score distributions, pointing out a highly variable behavior of this tool, potentially depending on its algorithm and, specifically, on the DNA secondary structure elements library.

These observations, together with the metrics distributions (Fig. S1), sustain the hypothesis that 3dDNA has a binary behavior: it either produces excellent models very close to the experimental structure or fails in the task, since not only the atoms’ positions but also the base pair patterns are not respected. This is an important element that has to be taken into account when choosing the tool to use to generate an initial ssDNA 3D structure: indeed, depending on the kind of expected structure and its further exploitation, in some cases, it would be more appropriate to use a tool for which it is known that the produced model will not be excellent, but it will neither be very far from the most probable experimental structure, as in the case of Vfold3D or SimRNA.

The Vfold3D failure to model 16 ssDNAs out of the 97 ssDNAs likely originates from its inability to find compatible motifs that meet the base pair constraints in the experimental secondary structure. This may especially occur when a structure contains only a single isolated base pair interaction. Indeed, if, instead of providing the experimental secondary structure constraints as an input, the whole Vfold procedure is applied (i.e., the use of Vfold2D for the prediction of the secondary structure followed by the modeling of the 3D structure prediction with Vfold3D), Vfold returned a model for 15 out of 16 sequences (Table S8 and Supplementary Materials). Nevertheless, these models are based on a Vfold2D-predicted secondary structure that does not fully correspond to the experimental one. As a consequence, all the modeled structures have INF scores ≤0.7, implying that the models fail to accurately reproduce the native interaction networks (Table S8 and Supplementary Materials). For example, in the case of a minidumbbell (PDB code 6M0B [48]), Vfold3D does not provide a 3D model when the experimental base pair constraints were given as input, but it was able to predict a 3D structure when no constraints were added, and we let Vfold2D suggest a secondary structure (**.((..)).**), that differs from the experimental one (**(..)(..)**). The only ssDNA that remained unmodeled even when using the whole Vfold pipeline is a 24-nucleotide oligonucleotide composed of 8 GGA triplet repeats (PDB code 1OZ8) [49]. This particular type of repeat is known to adopt a wide variety of structures [50–54]. In the case of 1OZ8, it forms a distinct structural motif characterized by an intramolecular higher order tail-to-tail packing of parallel G4. This particular folding pattern likely contributes to the Vfold inability to generate either a 3D model or even a secondary structure for this ssDNA. In addition, the models produced by RNAComposer, SimRNA, and 3dDNA for 1OZ8 were very far from the peculiar experimental 3D structure, with RMSD values of 18.8, 14.7, and 21.5 Å, respectively, INF scores of 0.12 for all 3 methods, and GDT scores of 25.0, 30.7, and 27.0, respectively.

As previously mentioned, 3dDNA fails in modeling 2 ssDNAs, both being characterized by a very short length (7 nucleotides), likely because of the impossibility of building structural elements for such short sequences.

### The impact of the ssDNA length on the performance of the tools

**Fig. 3.**
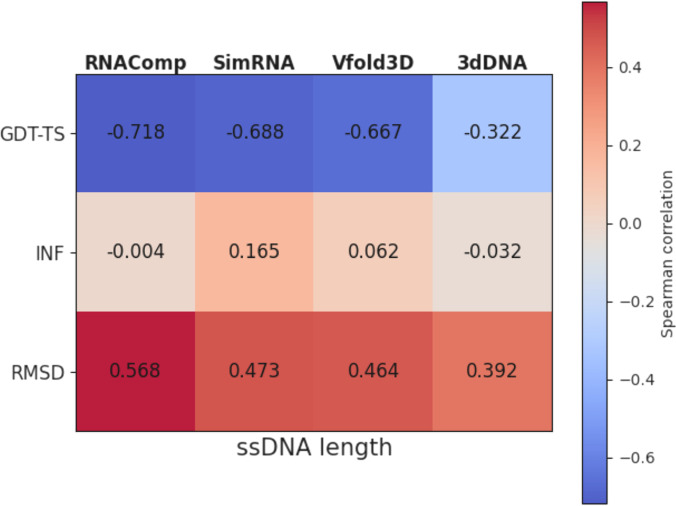
Correlation between the 3 structural metrics (RMSD, INF, and GDT_TS) across the 4 prediction tools (RNAComposer, SimRNA, Vfold3D, and 3dDNA) and the oligonucleotide length.

Having defined metrics thresholds to facilitate the investigation of the predictions’ quality, it is important to verify at which extent the obtained RMSD, INF, and GDT_TS values depend on the ssDNA length, and if differences between the 4 tools can be observed from this point of view. Therefore, the dependence of their prediction accuracy from the ssDNA length was assessed. With this aim, the Spearman correlation between the sequence length and the 3 structural metrics (RMSD, INF, and GDT_TS) was computed. The results reported in Fig. 3 show that the INF score does not correlate with the sequence length for any of the tools, indicating that the tools’ ability in reproducing the interaction network does not depend on the ssDNA size.

Conversely, as expected, the RMSD is moderately correlated with the sequence length for the indirect prediction tools and less for 3dDNA (RNAComposer: ρ = 0.57; SimRNA: ρ = 0.47; Vfold3D: ρ = 0.46; 3dDNA: ρ = 0.39). A positive correlation would indicate that the longer the sequence is, the higher the RMSD of the provided model is, and thus, the lower the structural accuracy is. However, the obtained correlation mostly depends on the heavy-atom RMSD obtained for very short oligonucleotides (<20 nucleotides). Indeed, as previously mentioned, a low RMSD is expected for short sequences, likely folding in simple and well-modeled structures. If these are removed, the weak correlation disappears, with a ρ = 0.16, −0.04, 0.13, and 0.18 for RNAcomposer, SimRNA, Vfold3D, and 3dDNA, respectively.

The GDT_TS correlation to the ssDNA length is more marked as compared to the RMSD one for the indirect prediction tools, with a mild negative correlation for RNAComposer (ρ=−0.72), SimRNA (ρ=−0.69), and Vfold3D (ρ=−0.67), indicating that the models obtained for long sequences have low GDT_TS, which are indicative of a poor quality structure as compared to the experimental one. In contrast, 3dDNA shows only a weak correlation (ρ=−0.32), indicating that its GDT_TS values are less dependent on sequence length.

### The issue of noncanonical motifs

Giving a closer look to the obtained results, it is possible to highlight some limits common to the 4 tools, with the most noticeable one being the modeling of ssDNAs containing G4 motifs. This issue is brought to the extreme by Vfold3D, which did not provide a model for 7 out of 26 G4-containing ssDNAs. Indeed, if these 26 structures are removed from the dataset, all the metrics improve (Table S7 and Supplementary Materials), with medians close to (RMSD) or above (INF and GDT_TS) the fixed thresholds, and the difference between the tools performances become slightly more significant (Table S6).

Moreover, for all the methods, none of the models containing a G4 motif had an RMSD ≤ 5 Å or an INF score ≥ 0.7. In addition, only 2 (2M8Z and 5CMX), 11 (2M91, 7CV3, 1I34, 1HAO, 148D, 7CV4, 6EVV, 5CMX, 2M8Z, 2M90, and 5VHE), 3 (2M91, 6EVV, and 2M8Z) and 4 (2M92, 5CMX, 2M8Z, and 2M91) structures predicted by RNAComposer, SimRNA, Vfold3D, and 3dDNA, respectively, provided a GDT_TS ≥ 45%. This slightly better performance suggested by the GDT_TS can be attributed to the fact that it uses a more nuanced approach to assess structural similarity by considering a range of distances between corresponding atoms in the predicted and reference structures and its stronger sequence-length dependence as compared to the other metrics.

**Fig. 4.**
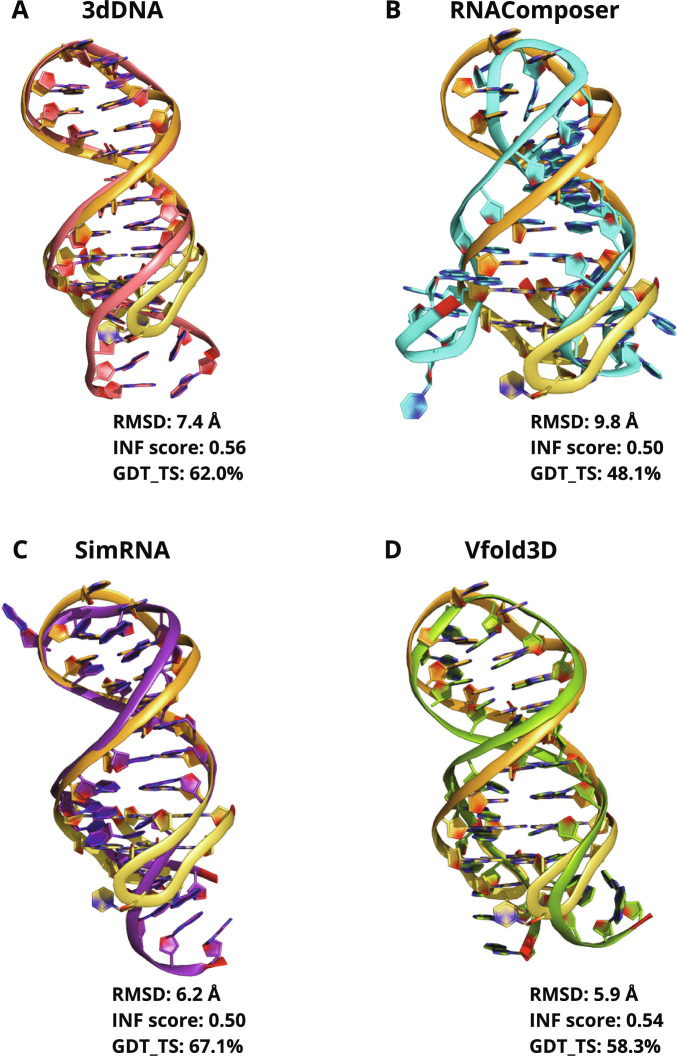
Alignment of the (A) 3dDNA, (B) RNAComposer, (C) SimRNA, and (D) Vfold3D predicted structures to the experimental structure of the 2M8Z ssDNA. The alignment has been realized by focusing on the only hairpin motif phosphate backbone. The experimental structure hairpin and G-quadruplex are colored orange and yellow, respectively.

Among the ssDNAs containing G4, 2M8Z is the one for which the 3 metrics provided the best results. This ssDNA is composed of 27 nucleotides organized in 2 distinct regions: a hairpin and a G4 formed by 2 G-quartets (Fig. 4). RNAComposer generated a model with a heavy-atom RMSD of 9.9 Å, a total INF score of 0.51, and a GDT_TS of 48.1%; the SimRNA model has a heavy-atom RMSD of 6.2 Å, a total INF score of 0.51, and a GDT_TS of 67.1%; Vfold3D globally provided the best model, with a heavy-atom RMSD of 6.0 Å, a total INF score of 0.55, and a GDT_TS of 58.3%. Finally, 3dDNA generated a model with a heavy-atom RMSD of 7.4 Å, a total INF score of 0.56, and a GDT_TS of 62.0%. Nevertheless, the acceptable performances obtained for this structure can be mainly attributed to the accurate prediction of the hairpin region. Indeed, when the G4 motif is excluded from the metrics computation, the INF scores improve to 1.0 for all 4 tools. Likewise, the RMSD values drop well below 5 Å, reaching 3.4, 2.8, 2.7, and 1.5 Å for RNAComposer, SimRNA, Vfold3D, and 3dDNA, respectively. The GDT_TS scores also improve (68.3%, 88.3%, 86.7%, and 91.7% for RNAComposer, SimRNA, Vfold3D, and 3dDNA, respectively) highlighting a generally higher predictive accuracy in the absence of the G4 motif. Figure 4 illustrates the quality of the alignment of the hairpin predicted loop when the G4 motif is ignored. These results indicate that all 4 tools accurately model the stem/loop motif, in contrast to their poor performance on the G4 motif. Indeed, no evidence of the formation of G4 is observed, and not even a partial rearrangement of the guanines into a quartet-like structure typically associated with G4 can be found. The same observations are applicable to all the G4-containing structures: none of the tools provided models where even a glimpse of G4 motif could be seen, independently from the type of the G4 motif. Indeed, even parallel G4, which can be found also in RNA structures, could not be modeled by the different tools. This is also true for G4-containing structures that were experimentally resolved in aqueous solution in the free state, where the G4 should therefore be the most stable conformation. In addition, it was not possible to find any G4-like structure in the ensemble of suboptimal conformations produced by the different tools (Table S13).

The results obtained for the G4-containing structures are not surprising: this motif is known for its intricate topology and stability, with hydrogen bonding on both the Hoogsteen and the Watson–Crick faces, and the presence of monovalent cations to stabilize the structure. The inability to explicitly include ions in the prediction process, together with the atypical base pair characterizing this motif, may explain the poor performances of the selected prediction tools and the failure to accurately capture these interactions. Potentially, the inclusion in the prediction algorithms of a learning step including, among all the data, those about G4 motifs would be an advantage when dealing with these structures. This can be proved by considering the potential performances of AlphaFold3 (AF3) [55] predicting this kind of motif. Although we could not include AF3 in this benchmark because most of the dataset we used is part of the AF3 training set and, therefore, all the ssDNAs will be well modeled, a recent work by Ochoa and Milam [56] showed the promising results produced by AF3 in predicting G4 motifs. In addition, we tested it for the prediction of the structure of a series of aptamers we recently selected against the *Borrelia burgdorferi* CspZ protein [57]. Some of these DNA aptamers were experimentally shown to fold into a parallel G4. This folding is correctly predicted by AF3 by adding an adequate number of K^+^ ions. Although the predicted reliability is low (Fig. S2), the discrepancy between confidence metrics and structural accuracy has already been observed [56].

Similarly to G4, intramolecular i-motifs are not correctly handled by the 4 tools because of their peculiar structural organization. This kind of structure can be found within an ssDNA with a characteristic sequence of the type $C_{n}X_{1}C_{n}X_{2}C_{n}X_{3}C_{n}$, where $C_{n}$ represents a consecutive run of cytosines and *X* denotes loop regions. This ssDNA folds back on itself so that the 4 cytosine stretches align in parallel and form 2 intercalated antiparallel duplexes. The structure is stabilized by hydrogen bonds between hemi-protonated cytosines (cytosines capable of capturing an additional proton) and adjacent cytosines, and its stability strongly depends on the pH [58]. The only structure having this motif found in the PDB database and not containing modified nucleotides is 8AYG [59]. This corresponds to a region of the insulin promoter and consists of a 31-nucleotide sequence enriched in cytosines, enabling it to fold into an i-motif structure [59]. For this ssDNA, none of the selected tools were able to generate an accurate model, with Vfold3D failing to produce any model at all. Because of the impossibility of extensively testing the tools’ performances for this particular motif, these results need to be handled with caution. However, as hypothesized for the G4 motif, the fragment libraries and the models on which the different tools are developed lack the information of these peculiar motifs. In addition, both of them are significantly affected by the presence of cations, which are not explicitly included by the 4 tools. Finally, since the i-motif has not been observed in RNA structures, the indirect prediction tools are further disadvantaged.

### Dealing with oligonucleotides flexibility

Although the conformational freedom of an ssNA can be taken into account by the different algorithms during the steps preceding the final structure generation (e.g., SimRNA uses Monte Carlo simulations at this scope), the ssNA structure prediction tools will provide one or, if specified, a limited number of consensus solutions. This might result in predictions that do not fall within the acceptance range for the chosen metrics.

Therefore, it is not surprising that, in addition to the failure in predicting the i-motif and G4 motifs, the 4 selected prediction tools struggle to different extents when the flexibility of ssDNA is particularly significant. This is the case of structures containing multiple-way junctions, long unpaired extremities, and long internal loops or bulges.

To gain deeper knowledge about the performances related to ssDNA flexibility, the models obtained for flexible ssDNA motifs whose structure has been resolved in a free form were initially analyzed.

Moreover, multiple-way junctions can be particularly flexible motifs, because of the potentially different relative orientation of the different branches. As a consequence, the quality of the produced model is variable. Within the built dataset, 4 ssDNAs possessing a multiple-way junction can be found (1EZN, 1SNJ, 2F1Q, and 3HXO). Except for 3HXO, their structures have been determined in the free state. Interestingly, 3dDNA produced highly accurate models, with RMSD values ranging from 0.64 to 1.23 Å, INF scores between 0.84 and 0.93, and GDT_TS values spanning from 93% to 99%. In contrast, the 3 indirect methods provided models with a heavy-atom RMSD ≥ 7 Å, a GDT_TS < 45%, and INF scores close to the fixed threshold (0.65 to 0.79). This indicates that the indirectly obtained models acceptably reproduce the base pair pattern, although the spatial orientation of the multiple-way junction is incorrect. Likely, indirectly obtained ssDNA multiple-way junction models will hardly reach the experimental junctional torsions without an intensive structural optimization, such as explicit solvent MD simulations.

**Fig. 5.**
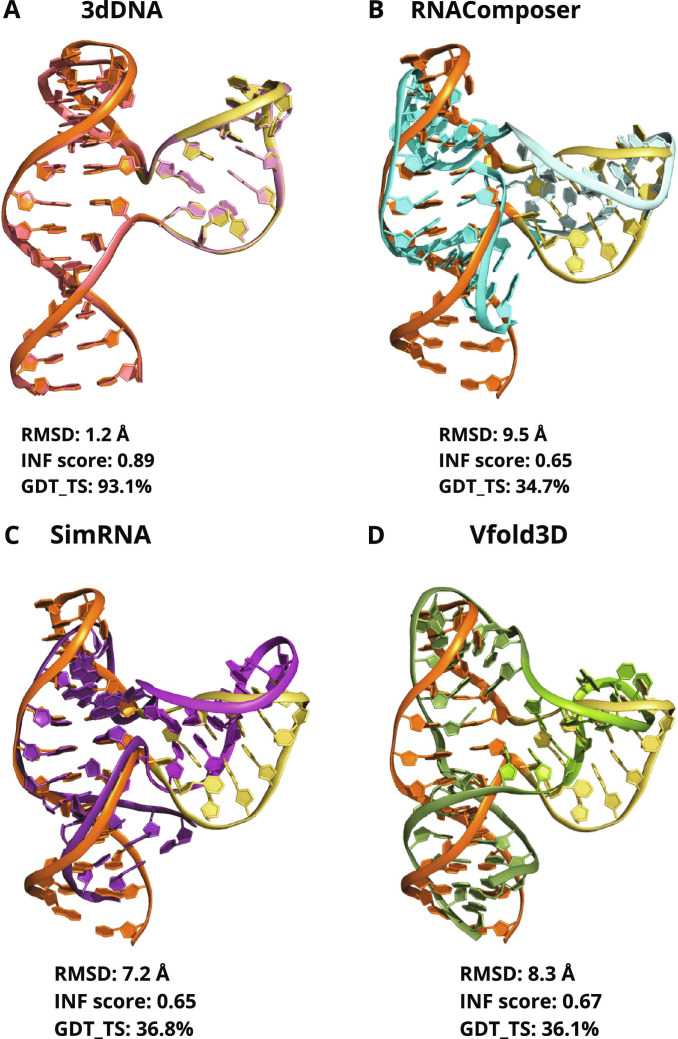
Alignment of the (A) 3dDNA, (B) RNAComposer, (C) SimRNA, and (D) Vfold3D predicted structures to the experimental structure of the 1SNJ ssDNA. The flexible hairpin is depicted in light pink, light cyan, purple, and light green for predicted structures, respectively, and in yellow for the experimental structure. Only the first conformer of the NMR structure is depicted. The heavy-atom RMSD from the first NMR conformer, the INF score, and the GDT_TS are reported for each model.

This observation can be illustrated by the structure with the PDB code 1SNJ [60], corresponding to a 3-way junction ssDNA resolved by solution NMR. The 12 submitted conformers for this structure indicate a potential flexibility located at the major hairpin (G18 to T29, in yellow in Fig. 5A). For this structure, 3dDNA produced a good model, with a heavy-atom RMSD of 3.7 Å, an INF score of 0.79, and a high GDT_TS value of 81.2%. The 3-way junction motif was accurately predicted, suggesting that this tool is capable of handling ssDNA containing this kind of motif. In contrast, RNAcomposer, Vfold3D, and SimRNA provided models that do not satisfy the requirements for any of the metrics with a heavy-atom RMSD of 9.6, 8.3, and 7.3 Å, respectively; an INF score of 0.66, 0.67, and 0.66, respectively; and a GDT_TS of 34.7%, 36.1%, and 38.2%, respectively (Fig. 5B to D). However, it can be observed that the main issue comes from the wrong orientation of the main hairpin (in yellow in Fig. 5) as compared to the experimental structure. This causes a distortion of both the hairpin involving the 5′ and 3′ ends and the second hairpin. Thus, although the experimental secondary structure is respected, alterations of the interactions within the ssDNA are observed, as indicated by the total INF score.

It is noteworthy that the analysis of the suboptimal solutions provided by the different tools did not allow the observation of any improvement in the prediction quality (Table S10), suggesting that a more intense exploration of the conformational space of highly flexible ssDNA is required to correctly sample all the possible orientations.

Another source of flexibility is represented by the presence of long loops or bulges (>4 nucleotides), which are found in 14 out of the 97 ssDNAs, 11 of which have been structurally resolved in complex with a protein. For what concerns the 3 ssDNA in a free state (1NGO, 1NGU, and 1EN1), the results are variable for the considered tools as a function of the structural complexity of the ssDNA: 1NGO, characterized by a long hairpin with an apical loop of 5 nucleotides, is the best predicted, with just the heavy-atom RMSD of the models obtained by the indirect tools being slightly above the fixed threshold (5.40, 6.04, and 5.17 Å, for RNAcomposer, SimRNA, and Vfold3D, respectively) (Fig. S3). Conversely, the ssDNA with the PDB code 1EN1 is the worst modeled among the ssDNAs of this group. This ssDNA is composed of 18 nucleotides, organized in a simple hairpin motif with a long loop of 5 unpaired nucleotides, a free extremity of 4 nucleotides, and an additional bulge. Overall, none of the methods achieved satisfactory performance for this structure, with heavy-atom RMSD between 6.84 Å (RNAcomposer) and 9.66 Å (SimRNA), INF scores between 0.70 (RNAcomposer) and 0.51 (SimRNA), and GDT_TS between 51.4% (RNAcomposer, SimRNA, and 3dDNA) and 50.0% (Vfold3D). RNAComposer was the only tool able to capture the correct interaction network, successfully predicting both the bulge and the canonical Watson–Crick base pairs. In contrast, the other methods, including 3dDNA, failed to reproduce this motif. Furthermore, all tools struggled to accurately model the free extremity, which is expected given its high flexibility and the lack of structural constraints, making its spatial positioning inherently difficult to predict (Fig. S4).

In this context, the difficulty of orienting a long loose ssDNA fragment is expected, and since, for this region, there are no structural constraints, it is freely moving, and except when another macromolecule, such as a protein, is present, it only makes interactions with the solvent.

### Structure prediction of ssDNAs in complex with a protein

The interaction with a binding partner, such as a protein, can affect the relative stability of the metastable conformations characterizing the ssDNAs accessible conformational space. Therefore, the analysis of the *in silico* predicted models of ssDNA interacting with a protein can provide interesting insights into the potential exploitation of these tools when designing and engineering ssDNAs for biotechnological applications. At this scope, in the built dataset, 49 ssDNA structures in complex with a protein were included. Among these, 8 contain a G4 motif, and the remaining are mostly characterized by motifs determined by canonical base pairs, such as hairpins with or without bulges and/or internal loops.

First of all, the analysis of the obtained models interestingly showed that the state of the ssDNA has a minor role on the prediction performances of the different tools. Indeed, the median values obtained for the different metrics on the subset of ssDNA in complex with a protein present are equivalent to or slightly better than those obtained on the whole dataset and those computed for the ssDNA in the free state (Table S11). In the light of this, an ssDNA that has been experimentally resolved in complex with a protein does not represent *per se* an issue for the prediction of its 3D structure. The only limit encountered in this context is related to the presence of noncanonical motifs, the oligonucleotides’ flexibility, and the interaction of the proteins with ssDNA highly flexible regions, such as loose ends, loops, and bulges. Indeed, Fig. 2 shows that the 4 prediction tools are able to provide good quality predictions based on the considered metrics for ssDNAs experimentally available in complex with a protein. For example, for 6 (4KB0, 2VHG, 2A6O, 6FK4, 1UUT, and 4KB1) out of the 49 ssDNA–protein complexes present in the dataset, RNAcomposer, SimRNA, Vfold3D, and 3dDNA provided models satisfying the threshold values set for the 3 considered metrics (Table S3). Notably, the ssDNAs belonging to these structures are folded into a quite simple hairpin, with, in some cases, a bulge of 1 or 2 nucleotides (4KB0, 2VHG, 2A6O, and 4KB1). These ssDNAs are, therefore, quite stable, thanks to 5 or more canonical base pairs. More importantly, most of them interact with their target protein through the grooves created by the stem or by their 5′–3′ extremities, while the apical loops are solvent-exposed, meaning that the conformation of the most flexible regions is not affected by the presence of the protein. 1UUT is the only structure where the apical loop is at the ssDNA–protein interface. However, it is made of only 3 nucleotides; thus, its conformational freedom is limited as compared to the previously discussed cases.

In addition, for 8 ssDNAs among the considered subset (1YTB, 4F41, 4F43, 6FK5, 6FKE, 5OND, 6SEI, and 6U82), the 4 tools provided globally good models, with scores within or close to the fixed thresholds. Conversely, among the ssDNA binding to a protein for which the different tools have medium to low modeling performances, we find 8 G4-containing ssDNAs (1HAO, 2M8Z, 2M90, 2M91, 2N21, 5CMX, 5VHE, and 6EVV), and 2 structures characterized by isolated base pairs (2A0I and 5F55), which have already been highlighted as challenging to model, independently from their state. The remaining ssDNAs of this subset are characterized by 11 structures containing long internal loops and/or bulges, 1 multiple-way junction, 11 ssDNAs with long unpaired ends, 2 pseudoknots, and 1 ssDNA organized in 2 independent hairpins.

For what concerns the structures possessing long loops and/or bulges (2EXF, 2JZW, 3WPD, 3WPG, 3WPH, 4I7Y, 2VJU, 3ZH2, 2VIC, and 3THW), the analysis of the produced models by the 4 tools is coherent to what observed for this kind of motifs found in ssDNA in the free state, confirming the hypothesis that only the degree of flexibility affects the modeling quality of the selected tools. Independently from the region involved in the interaction with the binding partner, the simpler is the structure, the better is the model, with 3dDNA globally showing better performances than the indirect tools.

The only ssDNA with a multiple-way junction resolved in complex with a protein is 3HXO, and it corresponds to an aptamer binding the von Willebrand factor (VWF) A1 domain and acting as an inhibitor of the VWF-platelet binding [61]. In this case, none of the tools are able to provide a good quality model. The heavy-atom RMSDs span from 5.73 Å (Vfold3D) to 9.20 Å (3dDNA), the GDT_TS ranges from 33.7% (RNAcomposer) to 39.4% (SimRNA), and the INF scores are between 0.62 (SimRNA) and 0.71 (RNAcomposer) (Fig. S5). Two of the aptamer arms are extensively interacting with the protein, and the VWF A1 domain might, therefore, stabilize a metastable conformation. This could explain the heavy difference of the modeling performances of 3dDNA for this kind of motif as a function of the state of the ssDNA, with excellent predictions obtained for the multiple-way junctions in the free state and a poor model obtained for 3HXO. Conversely, the results obtained for this aptamer by the indirect tools are coherent with those provided for the multiple-way junction structures resolved in the free state, with an incorrect orientation of the flexible arms in all cases and without a further worsening of the model due to the presence of the protein. Nevertheless, with this single example, it is not possible to unequivocally generalize the issue of modeling multiple-way junctions in complex with a protein, since it might be case-related.

The same carefulness has to be employed when making conclusions on the modeling of the only ssDNA of the whole dataset characterized by 2 independent hairpins connected by a short unpaired fragment (PDB code: 2N8A). In this case, all the tools provide predictions with high INF scores (≥ 0.79), indicating that the base pairs interactions are respected, but with poor RMSD and GDT_TS values, due to an incorrect spatial orientation of the nucleotides (Table S3). In this case, it is also difficult to conclude on the role played by the presence of the protein compared to that related to the intrinsic flexibility of this ssDNA.

In contrast, the subset of ssDNAs in complex with a protein contains a significant number of ssDNA with a long and, therefore, highly flexible unpaired extremity (1OSB, 1ZM5, 3C46, 3DSD, 3Q0A, 3Q23, 3Q24, 4ER8, 4FF1, 4HT4, and 5N2Q). In all the considered structures, this is always involved in the interaction with the binding partner, which contributes to the stabilization of a specific conformation.

**Fig. 6.**
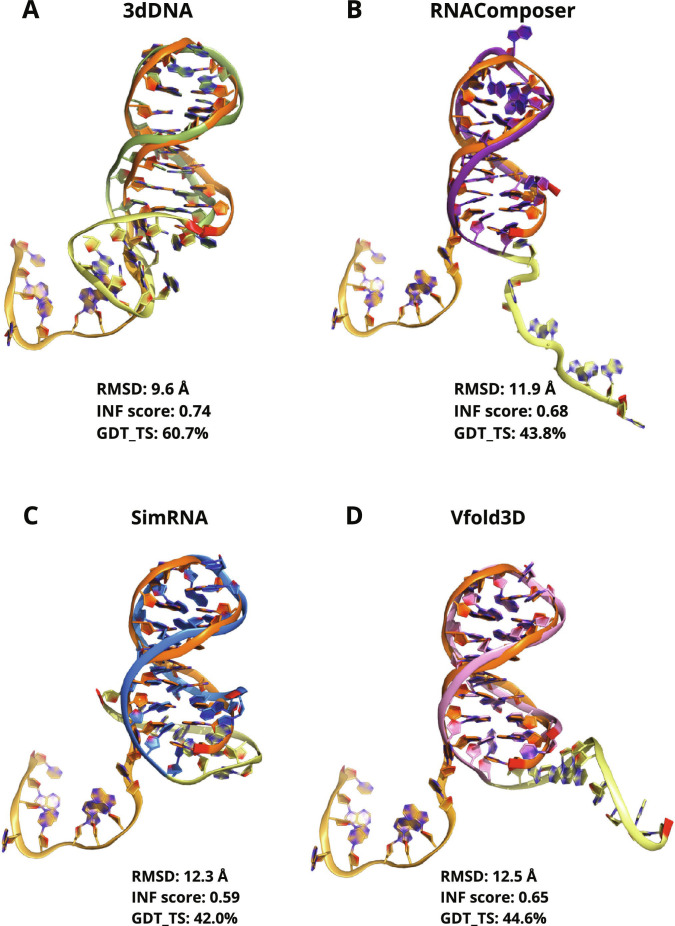
Alignment of the (A) 3dDNA, (B) RNAComposer, (C) SimRNA, and (D) Vfold3D predicted structures to the experimental structure of the 4HT4 ssDNA. The alignment has been realized by focusing on the only hairpin motif phosphate backbone. The experimental structure hairpin and the unpaired extension are colored orange and yellow, respectively.

This is the case of the x-ray-resolved structure of the *Staphylococcus aureus* nicking enzyme N-terminus relaxase–DNA complex involved in antibiotic resistance (PDB code: 4HT4 [62]). This complex contains a 28-nucleotide ssDNA composed of a stem loop region (nucleotides 1 to 19) followed by a long unpaired extension (nucleotides 20 to 28). Most of the nucleotides in this ssDNA participate in the interaction with the target protein, particularly those within the unpaired extension, which, therefore, might stabilize the observed position and orientation of this region in the x-ray structure. None of the 4 3D structure prediction tools is able to accurately reproduce the experimental conformation (Fig. 6A to D).

More precisely, RNAComposer, SimRNA, Vfold3D, and 3dDNA produced models with heavy-atom RMSD values of 11.9, 12.3, 12.5, and 9.6 Å; INF scores of 0.65, 0.59, 0.68, and 0.74; and GDT_TS values of 44.6%, 42.0%, 43.8%, and 60.7%, respectively. Together, these metrics indicate a global fold that remains substantially different from the experimental structure. It can be noted that the RMSD values deviate substantially from the predefined threshold as compared to the INF and GDT_TS scores. However, this difference mainly arises from the misplacement of the long and flexible unpaired extension, whereas the stem loop region is reasonably well predicted, resulting in comparatively higher INF and GDT_TS scores. Indeed, when this region is excluded from the structural comparison, the models generated by RNAComposer, SimRNA, Vfold3D, and 3dDNA show a markedly improved agreement with the experimental structure. The heavy-atom RMSDs decrease to 6.1, 5.4, 4.7, and 4.7 Å, respectively, while the INF scores increase to 0.73 (RNAcomposer) and 0.79 (SimRNA, Vfold3D, and 3dDNA). Consistently, the GDT_TS values also improve to 57.9%, 56.6%, 64.5%, and 64.5%, respectively. The same considerations can be done for the other ssDNA characterized by a long unpaired extremity, although for some of them (1OSB, 1ZM5, 3C46, 3Q0A, 3Q23, 3Q24, and 4FF1), 3dDNA provided good quality models. Given the high flexibility of loose ends, these results are expected, and the bad quality of the models can be safely damped: in solution, the loose ends will be freely moving, accessing a wide number of conformations, among which those predicted by the different tools might be found.

It is worth mentioning that, comparisons were also made between the 4HT4 experimental structure and ensemble of suboptimal conformations generated for each tool (Table S12), but we could not observe any significant improvement in the metrics. This is due to the limited exploration of the ssDNA conformational space performed by the modeling tools.

Finally, ssDNA–protein complex formation affects the ssDNA conformation depending on its degree of flexibility. Therefore, the modeling issues of the different tools are not related to the presence of a protein, but to the intrinsic conformational freedom of the ssDNA.

### Modeling long-distance interactions

**Fig. 7.**
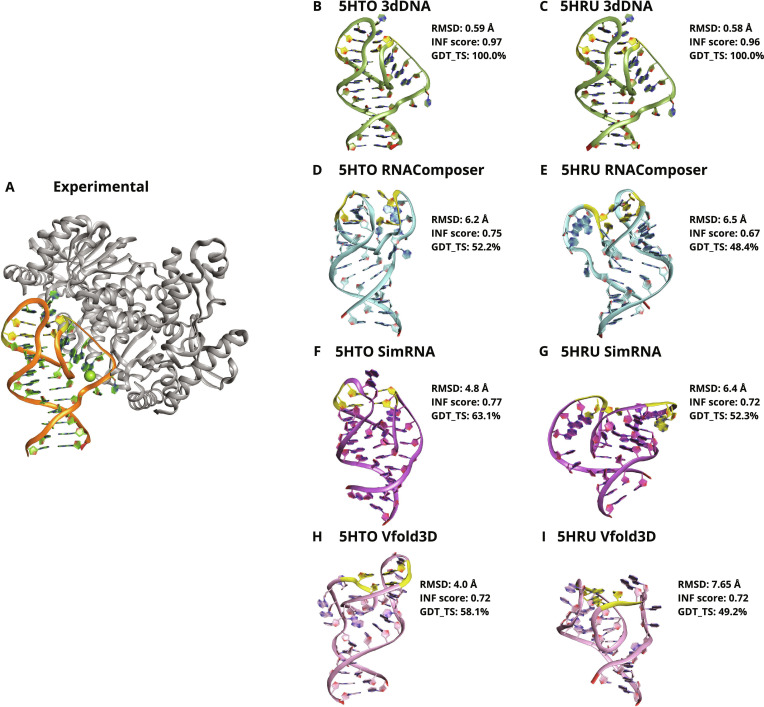
Experimental structure of pL1 in complex with *Plasmodium falciparum* lactate dehydrogenase (A) and predicted structures for 5HTO pL1 (B, D, F, and H) and 5HRU pL1 (C, E, G, and I) by 3dDNA, RNAcomposer, SimRNA, and Vfold3D. In (A), the experimental complex between the ssDNA aptamer and *P. falciparum* lactate dehydrogenase is reported. Only the first conformer of the solution NMR structure is shown. The nucleotides experimentally involved in the long-distance interactions are highlighted in yellow. The heavy-atom RMSD from the first NMR conformer, the INF score, and the GDT_TS are reported for each model.

Within the built dataset, 2 structures (5HTO and 5HRU [63]) contain a motif similar to a kissing hairpin pseudoknot, since 2 hairpins form 2 interactions (Fig. 7A). Both structures correspond to a DNA aptamer, called pL1, folded into a 3-way junction and targeting the *Plasmodium falciparum* lactate dehydrogenase. They differ solely for their crystallization conditions and for a length difference of 2 nucleotides, since the longest pL1 crystallized in 5HTO has an additional T at 5′ and an additional A at 3′. Together with the intrinsic flexibility of the 3-way junction structure, the presence of a long-distance interaction, which is also located at the interface with the lactate dehydrogenase, might represent an additional challenge, similar to what was previously observed for the flexible ssDNAs in complex with a protein.

In this case, 3dDNA is the only tool that provides a model very close to the experimental one for both structures: 5HTO and 5HRU with a heavy-atom RMSD of 0.59 and 0.58 Å and an INF score of 0.97 and 0.96, respectively. Regarding GDT_TS, both models have scores of 100%. Because of these astonishingly good results, we wonder to which extent the 2 PDB structures were excluded by the algorithm during the modeling as we specified in the input form. With 5HTO and 5HRU being the only available structures for the pL1 aptamer, to verify if the exclusion of the related experimental structure was taken into account during the prediction, we performed the 3dDNA modeling of both structures without the exclusion of the related PDB structures during the process. The obtained 5HTO and 5HRU models showed a heavy-atom RMSD as compared to the experimental structure of 0.37 and 0.20 Å, respectively (Fig. S6). This minor RMSD improvement indicates that the exclusion of the 2 PDB structures is taken into account, and that 3dDNA is able to correctly predict the long-distance interaction just based on the provided secondary structure.

The 3 indirect prediction tools successfully provided models for both structures, where the 2 kissing loops interact, suggesting that they are able to deal with long-distance interactions. Nevertheless, looking at the metrics, a trivial evaluation of the prediction performances concerning this particular case is not possible considering the models obtained by RNA 3D structure prediction tools. Indeed, if we consider the heavy-atom RMSD as compared to the experimental structure, the performances are acceptable only for SimRNA and Vfold3D and only for the pL1 contained in the 5HTO complex, since in the other cases, an RMSD > 5 Å is obtained (Table S3 and Fig. 7). When focusing on INF scores, all the tools perform well (INF > 0.7) on both structures, except for RNAcomposer, which provides a model for 5HRU with an INF score of 0.67. However, this score is close to the fixed threshold value and also to the scores obtained by the other tools (5HRU: 0.72 [SimRNA], 0.72 [Vfold3D]; 5HTO: 0.75 [RNAcomposer], 0.77 [SimRNA], 0.72 [Vfold3D]), which means that the 3 prediction tools are globally equally able to reproduce the nucleotides interactions found experimentally. Finally, by looking at the GDT_TS, all the tools provided models with a GDT_TS above the fixed threshold, though in all cases, the 5HTO aptamer was better modeled as compared to the 5HRU aptamer.

It should be kept in mind that the 4 tools obtained the nucleotides sequences together with the experimental ssDNA secondary structure as input. Except for 3dDNA, the sole models close to the experimental structure, all metrics considered, are those obtained by SimRNA and Vfold3D for the 5HTO pL1, even if Vfold3D provides a highly distorted orientation of the 2 hairpins forming the long-distance interaction. Conversely, RNAcomposer provided models not respecting the RMSD threshold, but the relative orientation of the kissing hairpins is closer to the experimental one as compared to the one produced by Vfold3D.

The indirectly obtained models of 5HRU pL1 are of lower quality as compared to those provided for 5HTO pL1. Indeed, although they show some sort of long-distance interaction between the 2 hairpins, the one provided by SimRNA does not involve the 2 experimentally interacting nucleotides, and the orientation of the kissing hairpins of the RNAcomposer and Vfold3D models is not in agreement with the experimental structure. Nevertheless, as previously mentioned, the *P. falciparum* lactate dehydrogenase might have a role in stabilizing the specific conformation observed for this flexible aptamer [63].

### Analysis of the secondary structures of the predicted models

In addition to the 3D structural comparison, an evaluation was conducted to assess how accurately the prediction tools preserved the secondary structure provided as input. For this purpose, the predicted secondary structures were compared with the secondary structures provided as input (see Materials and Methods, Table S14, and Figs. S7 and S8). To quantify the differences between the predicted and experimental 2D structures, the AptaMat metric [42] was used. This metric has already proven to be more sensitive to secondary structural changes as compared to the other commonly used metrics [42]. A threshold of 1.5 was fixed based on a previous study [43] to distinguish between accurate and inaccurate predictions, and all values ≤ 1.5 are considered close to the experimental secondary structure.

For this comparison, the structures containing G4 motifs were separated from the rest of the dataset, because the previously discussed issue in the prediction of this motif by the 3 tools implies that the secondary structure of the generated models will not match the experimental one (AptaMat score > 1.5).

Therefore, focusing on the sequences that do not contain a G4 motif or long-distance interactions (71 out of 97 ssDNAs), an overall good conservation of the 2D structure provided as an input was observed. More precisely, 3dDNA and RNAComposer achieved a perfect preservation of the experimental base pair pattern (AptaMat = 0) in 87.3% and in 88.7% of cases, respectively, while Vfold3D and SimRNA showed an AptaMat score of 0 in 64.8% and in 43.7% of the cases, respectively. If small deviations from the experimental secondary structure are accepted (i.e., AptaMat ≤ 1.5), 3dDNA provided 94.4%, RNAComposer 98.6%, SimRNA 78.8%, and Vfold3D 84.5% of the models with a secondary structure identical or highly similar to the experimental one.

The AptaMat score well correlates with the heavy-atom RMSD, with a Pearson’s correlation coefficient of ρ = 0.69, 0.62, 0.81, and 0.79, for RNAcomposer, SimRNA, Vfold3d, and 3dDNA, respectively (Fig. S9), indicating that, globally, a bad model does not respect the experimental secondary structure. Nevertheless, 32, 14, 21, and 8 models obtained by RNAComposer, SimRNA, Vfold3D, and 3dDNA, respectively, have an AptaMat score of 0 (Table S14) though their heavy-atom RMSD > 5 Å. However, these correspond to ssDNA previously identified for having highly flexible motifs (long loops and bulges, multiple-way junctions, and long unpaired extremities). In these cases, the global secondary structure can be respected while the spatial orientation of the nucleotides can deviate from the experimental one.

These results can be explained by the algorithm behind each tool: RNAComposer and 3dDNA build 3D models directly from a given 2D structure, strictly respecting the base-pair interactions. On the other hand, SimRNA and Vfold3D use more flexible, exploratory approaches, based on REMC and REMD simulations, respectively. Therefore, the latter can potentially modify the base pair pattern given as input. It should be noted that the satisfaction of the constraints of the experimental secondary structure does not imply that the final 3D model corresponds to the experimental structure. This underlines the importance of the modeling of loops and bulges, i.e., the fragments without any base pair. These, being highly flexible regions, benefit from the conformational sampling included in SimRNA and Vfold3D, as shown by the better performances of these tools as compared to RNAcomposer and 3dDNA.

Concerning the structures containing G4 motifs, as expected, neither 3dDNA, RNAComposer, nor Vfold3D produced models with an AptaMat score below 1.5. SimRNA generated only one structure with an AptaMat distance below this threshold, corresponding to the structure with the PDB code 2M92. This favorable score can be attributed to the accurate prediction of all the canonical base pairs in the model, which was not the case for the other structures. In addition, where no Watson–Crick base pairs were predicted and only Hoogsteen interactions were observed (e.g., 1HAO), the comparison was not possible. These cases are reported as None values (replaced by −1 in Fig. S8). This failure is mainly due to the absence of a properly formed G4 motif, which prevents its detection and, thus, the successful 2D reconstruction.

## Conclusion

ssDNAs are a highly interesting class of molecules, since they are involved in a plethora of biological processes and can be exploited for biotechnological applications. This is due to their ability to specifically and selectively recognize molecular targets, a feature strongly related to the 3D structures they can fold into. It is therefore clear that the understanding of the function and usage of these molecules would benefit from the analysis of their 3D structures. At this scope, many *in silico* prediction tools are available, and some of them have been evaluated with the CASP15 and CASP16 contests.

In this study, we assessed the performance for the prediction of ssDNA structures of 4 3D structure prediction tools: Vfold3D, SimRNA, RNAComposer, and 3dDNA. The first 3 tools were designed for RNA 3D structure prediction and have been selected based on their performance in the CASP15 and/or CASP16 challenges. 3dDNA was specifically developed to model DNA and ssDNA, although it relies on an algorithm initially developed for RNA structure prediction. Therefore, it allows to determine whether the new specific DNA modeling strategy confers a significant advantage in terms of structural accuracy compared with indirect RNA-based approaches adapted for DNA.

To perform this extensive benchmark, we built a dataset of 97 experimentally determined ssDNA structures, ranging from 7 to 53 nucleotides, retrieved from the PDB and the Nucleic Acid Database. This dataset includes a variety of structural motifs and ssDNAs in both free form and bound to proteins, providing a comprehensive assessment of structural predictions in different biological contexts. The predictive accuracy of these methods has been evaluated using several structural assessment metrics, including heavy-atom RMSD, GDT_TS to assess overall structural similarity, and the total INF score to evaluate the accuracy of predicted molecular interactions. The results obtained for RNAComposer, SimRNA, and Vfold3D are in agreement with those reported for the CASP15 and CASP16 contests, since they showed a moderate accuracy in predicting ssDNA structures. In contrast, 3dDNA shows overall better performances with an average 50% success rate, although it still exhibits limitations for certain structural motifs and highly flexible regions. Indeed, 3dDNA outperforms RNAComposer, SimRNA, and Vfold3D, with a median heavy-atom RMSD of 3.2 Å compared to 7.4, 6.8, and 6.6 Å, respectively, when evaluated against the experimental reference structures. Similarly, 3dDNA achieves a higher median INF score (0.84 vs. 0.65, 0.61, and 0.69) and a higher median GDT_TS value (77.5% vs. 48.4%, 53.1%, and 54.4%). However, this difference is much less dramatic if the overall distributions of the scores obtained for the different models are taken into account. Indeed, 3dDNA produced models either of extremely high quality (RMSD < 2 Å) or far from the experimental structure (RMSD > 7 Å). In addition, Vfold3D and 3dDNA failed to generate models for 16 and 2 out of the 97 structures, respectively. The missing Vfold3D predictions include 7 G-quadruplexes and 8 short sequences, whereas the 2 missing 3dDNA predictions correspond to very short 7-nucleotide sequences. Overall, these results indicate that 3dDNA produces more accurate ssDNA models than the 3 RNA-based structure prediction tools evaluated.

For RNAComposer, SimRNA, and Vfold3D, satisfactory models are mainly obtained for relatively simple structures, such as single hairpin motifs or highly stable conformations, while their performance markedly decreases for structures containing highly flexible elements, including long loops or complex motifs. In contrast, 3dDNA predicts simple structures more accurately and, in addition, shows a better ability to handle certain complex and flexible structural motifs, such as multiple-way junctions, which likely explains its overall superior performance compared with the other tools.

The 4 tools share the same limits. First of all, they performed poorly in modeling complex motifs such as G4 and i-motif structures: Vfold3D failed to generate 7 out of the 26 G4 structures, and none of the models of the structures containing this motif had an RMSD ≤ 5 Å or an INF score ≥ 0.7. In contrast, 3 models (6EVV, 2M91, and 2M8Z) satisfy the GDT_TS threshold, and among them, model 2M8Z is particularly successful, likely due to its simpler G4 formed only by 2 G-quartets (instead of the typical three), as well as the presence of more conventional structural elements, such as hairpin regions, which facilitate more accurate prediction.

Furthermore, the selected tools struggle to accurately predict the structure of ssDNA molecules that are highly flexible, such as structures with long loops and bulges. Although SimRNA and Vfold3D try to take into account this flexibility by using methods like Monte Carlo simulations or REMD, they still generate only one or a few possible structures. As a result, the predicted models often do not fall within the acceptance range for the chosen metrics. The inclusion of suboptimal models produced by the different tools do not significantly improve the predictions, indicating that the RNAcomposer, SimRNA, and Vfold3D exploration of the ssNA conformational space is insufficient. Interestingly, for structures containing multiple-way junctions, 3dDNA provides models very close to the experimental structures, whereas the other tools struggle with this motif and fail to produce accurate models.

The dataset also contains 2 DNA aptamers called pL1 (PDB codes 5HTO and 5HRU) that have a distance interaction similar to a kissing loop pseudoknot. 3dDNA is the only tool that provides a model very close to the experimental one for both structures. Conversely, RNAComposer, SimRNA, and Vfold3D provided models showing a long-distance interaction for the 2 hairpins in contact in the experimental structure. However, only the RNAComposer and SimRNA models for the 5HTO pL1 successfully reproduced the correct relative orientation of the kissing hairpins, while all other models (i.e., including those for 5HRU from all 3 tools and the Vfold3D model of 5HTO) failed to match the experimental structure.

This study highlighted the better performances of a tool, 3dDNA, which includes specific DNA elements for the prediction, as compared to the currently used indirect RNA-based prediction tools. The latter can still be used for simple structures, such as hairpins with a limited number of unpaired nucleotides, but for all the other motifs, 3dDNA has to be preferred. However, it has to be underlined that this tool provides models that are either very close to the experimental structure, probably because of matches in the DNA elements library, or definitively far from it, when structural elements do not correspond. This might limit its applications and require additional modeling steps focused on the exploration of the ssDNA conformational space.

Finally, 2 main issues remain independently from the chosen tool, the first one being the modeling complex motifs such as G4 and i-motif structures, and the second one depending on the intrinsic flexibility of ssDNAs. The former limit could benefit from the inclusion of an artificial intelligence-based step during modeling, which would learn from all the available ssNAs structures, including G4. Conversely, the treatment of the high conformational variability of ssNAs remains more difficult to handle, since any 3D structure prediction tool, by definition, has to provide one or a limited number of final models.

In the light of these results, 3dDNA can represent, for the moment, the tool of choice to generate one potential conformation for an ssDNA. However, this kind of tools can only be used to provide an initial configuration, and the exploration of the conformational space of ssDNAs still relies on other methods, such as enhanced sampling molecular dynamics techniques, which efficiently allow to do it, taking also into account the environment, and to associate a folding free energy and an occurrence probability to each generated conformation. With these approaches, stable and metastable configurations can be generated and exploited to investigate the structural properties of ssDNA, predict complexes with proteins, or design new engineered ssDNAs.

## Data Availability

All data generated or analyzed during this study are available upon reasonable request from the corresponding authors. The obtained data (obtained models) are available upon request to the authors. Bash and Python scripts used to automatize and analyze the data of this study are available upon request to the authors.
