# Supplementary material for: Predicting Single-Stranded DNA Oligonucleotides 3D Structures: An Open Issue
Source: Comput Struct Biotechnol J. 2026 Jun 8;35(1):0127. doi: 10.34133/csbj.0127 (PMC13244125; doi:10.34133/csbj.0127)
Supplement: Supplementary 1 — Tables S1 to S14 Figs. S1 to S9 [file csbj.0127.f1.pdf]

Supplementary Information for: Predicting Single-Stranded DNA Oligonucleotides 3D structures : an open issue

Selma Bengaouer[1], Thomas Binet[1], Stéphane Octave[1], Séverine Padiolleau-Lefèvre[1], Bérangère Avalle[1*], Irene Maffucci[1*]

[1]Université de technologie de Compiègne, CNRS, UPJV, GEC, Compiègne, France.

*Corresponding author(s). E-mail(s): avalle@utc.fr; irene.maffucci@utc.fr;
Contributing authors: selma.bengaouer@utc.fr; ; stephane.octave@utc.fr; severine.padiolleau@utc.fr;

List of Tables

Table S1 Single Stranded DNA dataset. 3
Table S2 Parameters applied to SimRNA algorithm 6
Table S3 RMSD, GDT-TS and total INF scores for the models obtained by RNAComposer, SimRNA, Vfold3D and 3dDNA for the ssNAs considered in this study. 6
Table S4 Summary of the structures not predicted by Vfold3D and 3dDNA. 13
Table S5 Pairwise Wilcoxon signed-rank test results for RMSD, INF and GDT_TS metrics for the whole dataset. 13
Table S6 Pairwise Wilcoxon signed-rank test results for RMSD, INF and GDT_TS metrics for the dataset excluding G4 and i-motif structures. . 14
Table S7 Mean and median heavy atoms RMSD, total INF score and GDT_TS obtained for the RNAComposer, SimRNA, Vfold3D and 3dDNA on ssDNA models. 15
Table S8 Summary of the structures predicted by the whole Vfold algorithm 16
Table S9 Metrics scores for the 15 predicted structures by the whole Vfold algorithm 16
Table S10 Comparison of 1SNJ suboptimal structures predicted by RNAComposer, Vfold3D and SimRNA 17

Table S11 Median heavy atoms RMSD, total INF score, and GDT_TS obtained for the RNAcomposer, SimRNA Vfold3D, and 3dDNA models in complex with a protein, in the free state, or both, including, excluding or focusing on G4-containing structures. 18
Table S12 Comparison of suboptimal structures predicted for 4HT4 19
Table S13 Heavy atoms RMSD values (Å) of suboptimal structures containing G4 motif generated by the four tools RNAComposer, SimRNA, Vfold3D and 3dDNA 20
Table S14 AptaMat score computed between the experimental and the models secondary structures. 22

List of Figures

Figure S1 Distributions of heavy atoms RMSD, INF score, and GDT-TS obtained for the models provided by RNAComposer, SimRNA, Vfold3D and 3dDNA. 24
Figure S2 AlphaFold3-predicted 3D structure of an aptamer targeting Borrelia burgdorferi CspZ protein. 25
Figure S3 Alignment of the 3dDNA, RNAComposer, SimRNA, and Vfold3D predicted structures to the experimental structure of the 1NGO ssDNA. 26
Figure S4 Alignment of the 3dDNA RNAComposer, SimRNA, and Vfold3D predicted structures to the experimental structure of the 1EN1 ssDNA. 27
Figure S5 Alignment of the experimental, 3dDNA, RNAComposer, SimRNA, and Vfold3D predicted structures to the experimental structure of the 3HXO ssDNA. 28
Figure S6 Alignment of the predicted 5HRU and 5HTO structures generated by 3dDNA without excluding their corresponding experimental structures. 29
Figure S7 AptaMat scores for the oligonucleotides secondary structures excluding G4 motifs. 30
Figure S8 AptaMat scores for the oligonucleotides secondary structures containing G4 motifs. 31
Figure S9 Correlation between AptaMat scores and RMSD values of the 3D structures predicted by RNAComposer, SimRNA, Vfold3D and 3dDNA. 32

Table S1: Single Stranded DNA dataset. Secondary structures are represented using dotbracket notation, where base pairs are denoted by matching opening and closing brackets, unpaired nucleotides by dots, and higher-order interactions with matching opening and closing square-brackets or braces. Following the Eltetrado notation, guanines involved in G-quadruplexes are also noted with sequential brackets and square-brackets.

PDB	Method	Size	G4	G4 type	Pseudoknot	Complex	i-motif	2D Structure
1AC7	NMR	16	No	—	No	No	No	((((((....))))))
1B4Y	NMR	30	No	—	No	No	No	..........((((((....))))))....
1BJH	NMR	11	No	—	No	No	No	((((...))))
1D16	X-RAY	16	No	—	No	No	No	((((((....))))))
1ECU	NMR	19	No	—	No	No	No	((((((((...))))))))
1EN1	NMR	18	No	—	No	No	No	((((((....)))))..)
1EZN	NMR	36	No	—	No	No	No	(((((((((((..))))((((..))))..)))))))
1JVE	NMR	27	No	—	No	No	No	((((((((((((...))))))))))))
1KR8	NMR	7	No	—	No	No	No	((...))
1LA8	NMR	13	No	—	No	No	No	(((((...)))))
1NGO	NMR	27	No	—	No	No	No	(((((((((((.....)))))))))))
1NGU	NMR	27	No	—	No	No	No	((((..(((((.....)))))..))))
1OSB	X-RAY	25	No	—	No	Yes	No	..(((((....)))))).........
1P0U	NMR	13	No	—	No	No	No	(((((...)))))
1PQT	NMR	7	No	—	No	No	No	((...))
1SNJ	NMR	36	No	—	No	No	No	(((((((((((..))))((((..))))..)))))))
1UUT	X-RAY	15	No	—	No	Yes	No	((((((...))))))
1XUE	NMR	17	No	—	No	No	No	((...((...))...))
1YTB	X-RAY	29	No	—	No	Yes	No	((((((((((((.....))))))))))))
1ZHU	NMR	10	No	—	No	No	No	..((...)).
1ZM5	X-RAY	25	No	—	No	Yes	No	..(((((....)))))).........
2A0I	X-RAY	10	No	—	No	Yes	No	....(..)..
2A6O	X-RAY	22	No	—	No	Yes	No	((((((((....)))).)))).
2EXF	NMR	14	No	—	No	Yes	No	..(((.....))).
2F1Q	NMR	42	No	—	No	No	No	(((((((((((..)))(((..)))((((..))))))))))))
2JZW	NMR	14	No	—	No	Yes	No	..(((.....))).
2K71	NMR	8	No	—	No	No	No	((....))
2L5K	NMR	23	No	—	No	No	No	(((...((((...))))...)))
2LO5	NMR	12	No	—	No	No	No	((((...).)))
2LO8	NMR	10	No	—	No	No	No	(((...).))
2M8Y	NMR	15	No	—	No	No	No	((((((...))))))
2N8A	NMR	45	No	—	No	Yes	No	(((((((((....))))))))).((((((((((..))))))))))
2VHG	X-RAY	24	No	—	No	Yes	No	((((((((....)))).))))...
2VIC	X-RAY	26	No	—	No	Yes	No	....((((((((.....)))).))))
2VJU	X-RAY	35	No	—	No	Yes	No	((..((((((((....)))).))))......))..
3C46	X-RAY	21	No	—	No	Yes	No	.......(((((...))))).
3DSD	X-RAY	23	No	—	No	Yes	No	.(((((((....)))))))....
3HXO	X-RAY	40	No	—	No	Yes	No	.(((((..(.(((...))))...(((.....))).)))))
3THW	X-RAY	53	No	—	No	Yes	No	(((((((((((((((((((((((...))))))))))....)))))))))))))
3Q0A	X-RAY	20	No	—	No	Yes	No	.......(((((...)))))
3Q23	X-RAY	21	No	—	No	Yes	No	.......(((((...))))).
3Q24	X-RAY	22	No	—	No	Yes	No	........(((((...))))).

3WPD	X-RAY	10	No	—	No	Yes	No	((.....)).
3WPG	X-RAY	11	No	—	No	Yes	No	((.....))..
3WPH	X-RAY	12	No	—	No	Yes	No	((.....))...
3ZH2	X-RAY	27	No	—	No	Yes	No	(((((.......((....))..)))))
4ER8	X-RAY	32	No	—	No	Yes	No	......(((((..(((((..)))))..)))))
4F41	X-RAY	32	No	—	No	Yes	No	(((((((((((((((..)))))))))))))))
4F43	X-RAY	32	No	—	No	Yes	No	(((((((((((((((..)))))))))))))))
4FF1	X-RAY	20	No	—	No	Yes	No	.......(((((...)))))
4HT4	X-RAY	28	No	—	No	Yes	No	.(((((((...)))))))..........
4I7Y	X-RAY	27	No	—	No	Yes	No	((((...(..(.....)..)..).)))
4KB0	X-RAY	18	No	—	No	Yes	No	((((((....)))))..)
4KB1	X-RAY	18	No	—	No	Yes	No	((((((....)))))..)
5F55	X-RAY	14	No	—	No	Yes	No	......(....)..
5GWL	NMR	8	No	—	No	No	No	(..)(..)
5GWQ	NMR	8	No	—	No	No	No	(..)(..)
5HRU	X-RAY	32	No	—	Yes	Yes	No	((((.....[[..(((((]]...)))))))))
5HTO	X-RAY	34	No	—	Yes	Yes	No	(((((.....[[..(((((]]...))))))))))
5N2Q	X-RAY	26	No	—	No	Yes	No	(((((((....))))))))........
5OND	X-RAY	9	No	—	No	Yes	No	((....).)
6FK4	X-RAY	16	No	—	No	Yes	No	..(((((...))))).
6FK5	X-RAY	14	No	—	No	Yes	No	(((((...))))).
6FKE	X-RAY	12	No	—	No	Yes	No	.((((...))))
6IY5	NMR	10	No	—	No	No	No	((...))...
6J37	NMR	8	No	—	No	No	No	(..)(..)
6M0B	NMR	8	No	—	No	No	No	(..)(..)
6M0C	NMR	8	No	—	No	No	No	(..)(..)
6SEI	X-RAY	32	No	—	No	Yes	No	.(((.(((((((((...)))))))..)).)))
6U82	X-RAY	38	No	—	No	Yes	No	((((((((((((((((...))))))))))...))))))
8AYG	X-RAY	31	No	—	No	No	Yes	...((((...[[[[...))))...]]]]...
148D	NMR	15	Yes	Antiparallel	No	No	No	([..[)...(]..])
1HAO	X-RAY	15	Yes	Antiparallel	No	Yes	No	((..)(...)(..))
1I34	NMR	20	Yes	Antiparallel	No	No	No	((....[[..)].....)].
1OZ8	NMR	24	Yes	parallel	No	No	No	(..)..(..)...(..)..(..).
2HY9	NMR	26	Yes	hybrid	No	No	No	...(([...)]...[[...)]]..
2KF8	NMR	22	Yes	Antiparallel	No	No	No	((....)[.....)(...)]..
2M8Z	NMR	27	Yes	Antiparallel	No	Yes	No	([..[)((((((...))))))(]..])
2M90	NMR	32	Yes	parallel	No	Yes	No	(((((((...))))))([.)].)([...)]
2M91	NMR	30	Yes	Antiparallel	No	Yes	No	((...)[.((((((...)))))).)(..)]
2M92	NMR	34	Yes	parallel	No	No	No	(([.)].(.))([.((((((...)))))).)]
2M93	NMR	32	Yes	parallel	No	No	No	..([.)].(((((...))))).([.)].
2N21	NMR	18	Yes	parallel	No	Yes	No	..([.)].([.)].
5CMX	X-RAY	30	Yes	Antiparallel	No	Yes	No	((((((.([..[)...(]..]).)))))).
5MTA	NMR	34	Yes	hybrid	No	No	No	..(([....)].(.......).[[)....]]..
5NYS	NMR	20	Yes	parallel	No	No	No	..([..)].([..)].
5VHE	X-RAY	24	Yes	parallel	No	Yes	No	.([.)].([.)]........
6EVV	X-RAY	26	Yes	Antiparallel	No	Yes	No	((((.(([..[)...(]..]))))))
6H1K	X-RAY	28	Yes	hybrid	No	No	No	((..(((...))).[[.)]...])
6SUU	NMR	32	Yes	parallel	No	No	No	.([.)]...([...........)]....

6T2G	NMR	32	Yes	parallel	No	No	No	.([.)]..([............)]....
6ZL2	NMR	36	Yes	parallel	No	No	No	...([.)]..([.)](((((((...)))))))
6ZL9	NMR	35	Yes	parallel	No	No	No	(((((((...)))))))([.)]..([.)]..
6ZTE	NMR	36	Yes	parallel	No	No	No	(((((((...)))))))([.)].([.)]....
7CLS	NMR	33	Yes	parallel	No	No	No	..([.(((((...)))))..)].([.)].
7CV3	NMR	27	Yes	hybrid	No	No	No	..(([.)](((....)))[[..)]]
7CV4	NMR	26	Yes	Antiparallel	No	No	No	(([..[)(((....)))(]....]))

Table S2: Parameters applied to SimRNA algorithm

Number of iterations	16 000 000
Trajectory recorded every n iterations	16 000
Initial temperature factor	1.35
Final temperature factor	0.9
Bonds weight	1
Angles weight	1
Tortions angles weight	0
$\eta + \theta$ angles weight	0.4

Table S3: RMSD, GDT-TS and total INF scores for the models obtained by RNAComposer, SimRNA, Vfold3D and 3dDNA for the ssNAs considered in this study.

PDB	Metrics	RNAComposer	SimRNA	Vfold3D	3dDNA
1AC7	RMSD (Å)	3.90	3.83	3.51	0.35
	INF	0.87	0.89	0.81	1.00
	GDT-TS (%)	73.43	75.00	73.43	100.00
1B4Y	RMSD (Å)	16.29	4.14	13.65	8.94
	INF	0.40	0.50	0.59	0.46
	GDT-TS (%)	40.84	76.56	47.50	45.84
1BJH	RMSD (Å)	3.93	3.83	3.10	2.57
	INF	0.89	0.60	0.83	0.86
	GDT-TS (%)	63.64	82.14	79.55	77.28
1D16	RMSD (Å)	5.83	8.90	8.76	3.44
	INF	0.85	0.37	0.36	1.0
	GDT-TS (%)	60.94	37.50	37.50	100.00
1ECU	RMSD (Å)	3.37	3.23	2.68	2.43
	INF	0.83	0.94	0.98	0.88
	GDT-TS (%)	73.69	78.84	81.58	92.10
1EN1	RMSD (Å)	6.84	9.66	7.90	8.32
	INF	0.70	0.51	0.65	0.55
	GDT-TS (%)	51.39	58.34	50.00	51.39
1EZN	RMSD (Å)	11.04	10.27	6.99	0.64
	INF	0.79	0.72	0.78	0.93
	GDT-TS (%)	32.64	36.00	43.75	99.31
1JVE	RMSD (Å)	5.35	4.23	3.99	1.0
	INF	0.81	0.78	0.91	1.00
	GDT-TS (%)	59.26	62.50	66.67	98.15
1KR8	RMSD (Å)	3.80	3.50	3.14	nc*
	INF	0.47	0.58	0.72	nc*
	GDT-TS (%)	75.00	89.28	92.86	nc*
1LA8	RMSD (Å)	3.56	4.30	4.28	0.35
	INF	0.86	0.68	0.73	0.92
	GDT-TS (%)	71.16	77.78	65.38	100.00
1NGO	RMSD (Å)	5.40	6.04	5.17	0.24
	INF	0.87	0.86	0.85	1.00
	GDT-TS (%)	51.85	54.42	60.18	100.00
1NGU	RMSD (Å)	7.38	7.72	7.19	0.29
	INF	0.69	0.83	0.76	0.98

Continue on next page

Table S3- *Continued from previous page*

PDB	Metrics	RNAComposer	SimRNA	Vfold3D	3dDNA
	GDT-TS (%)	41.67	46.67	41.67	100.00
1OSB	RMSD (Å)	8.28	8.27	6.62	2.88
	INF	0.65	0.54	0.49	0.83
	GDT-TS (%)	51.00	61.11	52.00	93.00
1P0U	RMSD (Å)	3.75	2.74	2.83	0.25
	INF	0.64	0.86	0.88	0.97
	GDT-TS (%)	69.23	83.33	73.08	100.00
1PQT	RMSD (Å)	3.80	3.51	3.39	nc*
	INF	0.47	0.67	0.63	nc*
	GDT-TS (%)	78.57	95.00	89.28	nc*
1SNJ	RMSD (Å)	9.57	7.29	8.32	1.23
	INF	0.66	0.65	0.67	0.89
	GDT-TS (%)	34.72	44.00	36.11	93.06
1UUT	RMSD (Å)	3.17	3.67	4.22	1.73
	INF	0.81	0.89	0.82	0.97
	GDT-TS (%)	65.00	93.75	73.33	85.00
1XUE	RMSD (Å)	3.82	4.44	6.97	0.79
	INF	0.65	0.70	0.44	1.00
	GDT-TS (%)	61.76	62.51	51.47	97.06
1YTB	RMSD (Å)	4.41	5.36	4.79	3.23
	INF	0.78	0.84	0.81	0.86
	GDT-TS (%)	63.79	57.35	59.48	85.34
1ZHU	RMSD (Å)	4.41	3.89	4.14	0.75
	INF	0.34	0.67	0.68	1.00
	GDT-TS (%)	62.50	79.17	67.50	97.50
1ZM5	RMSD (Å)	8.46	8.50	6.74	1.59
	INF	0.65	0.57	0.50	0.94
	GDT-TS (%)	46.00	61.11	44.00	97.00
2A0I	RMSD (Å)	5.08	6.76	nc*	0.41
	INF	0.63	0.17	nc*	1.00
	GDT-TS (%)	70.00	70.83	nc*	100.00
2A6O	RMSD (Å)	4.87	4.77	4.35	2.11
	INF	0.75	0.84	0.82	0.94
	GDT-TS (%)	61.36	67.86	72.73	92.05
2EXF	RMSD (Å)	4.28	3.91	4.32	3.95
	INF	0.70	0.70	0.69	0.58
	GDT-TS (%)	60.71	81.25	66.08	62.50
2F1Q	RMSD (Å)	10.27	12.57	11.67	1.06
	INF	0.72	0.75	0.69	0.84
	GDT-TS (%)	32.74	35.12	36.90	95.83
2JZW	RMSD (Å)	5.71	4.94	5.46	3.603
	INF	0.82	0.67	0.73	0.80
	GDT-TS (%)	48.21	75.00	55.36	62.50
2K71	RMSD (Å)	3.59	3.58	3.82	0.63
	INF	0.78	0.89	0.82	1.00
	GDT-TS (%)	84.38	83.34	78.12	100.00
2L5K	RMSD (Å)	6.73	5.72	4.20	0.63
	INF	0.72	0.83	0.68	0.93
	GDT-TS (%)	46.74	59.62	68.48	98.91

Continue on next page

Table S3- *Continued from previous page*

PDB	Metrics	RNAComposer	SimRNA	Vfold3D	3dDNA
2LO5	RMSD (Å)	4.75	4.63	4.50	2.17
	INF	0.70	0.78	0.75	0.87
	GDT-TS (%)	62.50	83.34	56.25	75.00
2LO8	RMSD (Å)	4.24	4.16	4.12	2.33
	INF	0.71	0.64	0.45	0.94
	GDT-TS (%)	62.50	71.43	70.00	80.00
2M8Y	RMSD (Å)	3.84	2.90	2.90	0.83
	INF	0.82	0.80	0.84	0.89
	GDT-TS (%)	66.67	81.82	75.00	100.00
2N8A	RMSD (Å)	11.10	12.74	8.62	10.47
	INF	0.84	0.79	0.82	0.90
	GDT-TS (%)	40.55	36.03	36.11	50.00
2VHG	RMSD (Å)	4.79	4.60	4.30	1.89
	INF	0.71	0.73	0.80	0.95
	GDT-TS (%)	61.45	67.19	64.58	83.34
2VIC	RMSD (Å)	6.26	5.37	4.68	1.89
	INF	0.78	0.70	0.68	0.97
	GDT-TS (%)	48.07	54.42	59.61	84.61
2VJU	RMSD (Å)	8.59	6.38	6.45	8.35
	INF	0.53	0.59	0.66	0.68
	GDT-TS (%)	38.57	60.87	50.71	56.43
3C46	RMSD (Å)	7.72	14.08	8.20	0.34
	INF	0.74	0.62	0.72	0.97
	GDT-TS (%)	48.81	57.36	55.95	100.00
3DSD	RMSD (Å)	8.32	8.56	9.09	6.39
	INF	0.80	0.68	0.73	0.84
	GDT-TS (%)	54.34	61.54	54.35	76.09
3HXO	RMSD (Å)	8.77	6.77	5.73	9.20
	INF	0.71	0.62	0.69	0.66
	GDT-TS (%)	33.75	38.33	36.25	34.38
3THW	RMSD (Å)	10.15	7.26	7.88	10.64
	INF	0.81	0.87	0.89	0.90
	GDT-TS (%)	41.51	32.50	41.98	55.19
3Q0A	RMSD (Å)	7.29	13.99	7.56	0.53
	INF	0.66	0.64	0.79	0.98
	GDT-TS (%)	48.75	60.00	62.50	100.00
3Q23	RMSD (Å)	7.64	13.60	8.07	0.82
	INF	0.80	0.69	0.72	0.97
	GDT-TS (%)	48.81	55.88	57.14	98.81
3Q24	RMSD (Å)	7.56	15.35	9.75	0.27
	INF	0.82	0.68	0.82	1.00
	GDT-TS (%)	46.59	55.88	50.00	100.00
3WPD	RMSD (Å)	4.30	4.70	3.51	4.06
	INF	0.56	0.42	0.74	0.57
	GDT-TS (%)	70.00	79.17	85.00	77.50
3WPG	RMSD (Å)	4.46	4.89	4.01	4.14
	INF	0.50	0.36	0.68	0.46
	GDT-TS (%)	75.00	78.57	84.09	70.45

Continue on next page

Table S3- *Continued from previous page*

PDB	Metrics	RNAComposer	SimRNA	Vfold3D	3dDNA
3WPH	RMSD (Å)	4.97	5.79	4.70	4.57
	INF	0.48	0.42	0.57	0.43
	GDT-TS (%)	72.92	78.12	79.17	70.83
3ZH2	RMSD (Å)	8.50	11.89	9.51	0.89
	INF	0.75	0.53	0.63	0.94
	GDT-TS (%)	37.04	29.76	44.44	95.37
4ER8	RMSD (Å)	8.21	8.18	6.81	6.05
	INF	0.66	0.75	0.70	0.80
	GDT-TS (%)	42.97	44.79	46.87	65.63
4F41	RMSD (Å)	6.82	5.84	5.72	1.18
	INF	0.80	0.91	0.92	0.96
	GDT-TS (%)	45.31	53.12	54.69	98.44
4F43	RMSD (Å)	7.02	6.16	5.53	0.86
	INF	0.77	0.92	0.89	0.97
	GDT-TS (%)	50.00	55.56	55.47	98.44
4FF1	RMSD (Å)	7.21	14.34	7.74	0.45
	INF	0.78	0.70	0.81	0.95
	GDT-TS (%)	51.25	61.67	60.00	100.00
4HT4	RMSD (Å)	11.93	12.26	12.48	9.64
	INF	0.65	0.59	0.68	0.74
	GDT-TS (%)	44.64	42.05	43.75	60.71
4I7Y	RMSD (Å)	8.65	8.94	9.70	13.55
	INF	0.51	0.38	0.45	0.37
	GDT-TS (%)	30.56	36.11	27.78	37.04
4KB0	RMSD (Å)	4.70	4.13	4.78	1.07
	INF	0.79	0.75	0.75	0.90
	GDT-TS (%)	58.33	79.17	68.06	97.22
4KB1	RMSD (Å)	4.59	3.90	4.40	0.70
	INF	0.81	0.75	0.79	0.90
	GDT-TS (%)	61.11	79.55	66.67	98.61
5F55	RMSD (Å)	9.94	11.68	nc*	8.54
	INF	0.50	0.27	nc*	0.35
	GDT-TS (%)	42.86	50.00	nc*	42.85
5GWL	RMSD (Å)	2.92	6.16	nc*	2.04
	INF	0.47	0.00	nc*	-1.00
	GDT-TS (%)	78.12	59.38	nc*	87.50
5GWQ	RMSD (Å)	5.69	5.76	nc*	2.51
	INF	0.65	0.00	nc*	-1.00
	GDT-TS (%)	50.00	56.25	nc*	81.25
5HRU	RMSD (Å)	6.48	6.38	7.42	0.58
	INF	0.67	0.72	0.72	0.96
	GDT-TS (%)	48.44	52.27	49.22	100.00
5HTO	RMSD (Å)	6.15	4.76	4.97	0.59
	INF	0.75	0.77	0.72	0.97
	GDT-TS (%)	52.21	63.05	58.08	100.00
5N2Q	RMSD (Å)	13.70	14.66	6.61	6.51
	INF	0.79	0.79	0.77	0.84
	GDT-TS (%)	45.19	56.66	57.69	66.35

Continue on next page

Table S3- *Continued from previous page*

PDB	Metrics	RNAComposer	SimRNA	Vfold3D	3dDNA
5OND	RMSD (Å)	3.88	4.55	nc*	1.51
	INF	0.75	0.59	nc*	1.00
	GDT-TS (%)	83.33	80.00	nc*	97.22
6FK4	RMSD (Å)	4.22	4.08	3.73	2.99
	INF	0.79	0.76	0.82	0.80
	GDT-TS (%)	68.75	81.25	85.94	87.50
6FK5	RMSD (Å)	4.40	3.57	3.37	2.21
	INF	0.61	0.81	0.82	0.92
	GDT-TS (%)	67.86	90.00	87.50	92.86
6FKE	RMSD (Å)	3.65	3.12	2.79	2.11
	INF	0.68	0.76	0.74	0.90
	GDT-TS (%)	70.83	93.75	93.75	87.50
6IY5	RMSD (Å)	7.77	4.04	6.45	5.59
	INF	0.89	0.60	0.32	0.73
	GDT-TS (%)	52.50	75.00	52.50	65.00
6J37	RMSD (Å)	2.39	5.49	nc*	1.72
	INF	0.47	0.33	nc*	0.67
	GDT-TS (%)	78.12	59.38	nc*	87.50
6M0B	RMSD (Å)	3.09	5.23	nc*	1.83
	INF	0.47	0.31	nc*	0.73
	GDT-TS (%)	71.87	56.25	nc*	87.50
6M0C	RMSD (Å)	4.33	6.03	nc*	2.02
	INF	0.53	0.13	nc*	0.85
	GDT-TS (%)	59.38	59.38	nc*	90.62
6SEI	RMSD (Å)	5.99	5.99	5.34	6.88
	INF	0.88	0.87	0.84	0.88
	GDT-TS (%)	53.90	48.87	62.50	50.78
6U82	RMSD (Å)	5.81	6.77	5.42	1.38
	INF	0.84	0.86	0.84	0.95
	GDT-TS (%)	52.63	41.35	52.64	98.69
148D	RMSD (Å)	8.84	6.51	9.26	14.25
	INF	0.00	0.00	0.00	0.00
	GDT-TS (%)	43.33	60.71	40.00	33.33
1HAO	RMSD (Å)	9.11	7.37	11.80	13.60
	INF	0.00	0.00	0.00	0.00
	GDT-TS (%)	43.33	53.57	35.00	35.00
1I34	RMSD (Å)	10.00	9.80	16.61	8.74
	INF	0.08	0.08	0.23	0.06
	GDT-TS (%)	33.75	50.00	35.00	31.25
1OZ8	RMSD (Å)	18.79	14.68	nc*	11.02
	INF	0.12	0.12	nc*	0.20
	GDT-TS (%)	25.00	30.68	nc*	30.21
2HY9	RMSD (Å)	20.00	12.78	23.54	13.47
	INF	0.26	0.26	0.21	0.13
	GDT-TS (%)	28.85	34.72	25.96	33.66
2KF8	RMSD (Å)	12.43	11.88	nc*	15.99
	INF	0.19	0.19	nc*	0.10
	GDT-TS (%)	40.91	35.71	nc*	32.96

Continue on next page

Table S3- *Continued from previous page*

PDB	Metrics	RNAComposer	SimRNA	Vfold3D	3dDNA
2M8Z	RMSD (Å)	9.88	6.25	5.99	7.46
	INF	0.51	0.51	0.55	0.57
	GDT-TS (%)	48.15	67.11	58.33	62.04
2M90	RMSD (Å)	10.53	12.88	14.95	12.40
	INF	0.55	0.55	0.65	0.56
	GDT-TS (%)	35.94	45.37	42.19	44.53
2M91	RMSD (Å)	10.38	8.89	9.02	10.51
	INF	0.55	0.55	0.59	0.59
	GDT-TS (%)	44.17	50.00	48.34	54.17
2M92	RMSD (Å)	11.66	12.12	13.38	13.38
	INF	0.45	0.45	0.66	0.47
	GDT-TS (%)	33.83	42.59	38.97	47.80
2M93	RMSD (Å)	12.39	10.26	12.86	10.35
	INF	0.44	0.44	0.61	0.49
	GDT-TS (%)	31.25	44.00	40.63	38.28
2N21	RMSD (Å)	14.14	10.29	nc*	14.99
	INF	0.21	0.21	nc*	0.37
	GDT-TS (%)	34.72	35.41	nc*	36.11
5CMX	RMSD (Å)	7.91	7.15	7.94	12.00
	INF	0.41	0.41	0.45	0.47
	GDT-TS (%)	45.00	55.00	44.17	46.66
5MTA	RMSD (Å)	18.79	11.07	18.31	19.08
	INF	0.21	0.21	0.25	0.09
	GDT-TS (%)	24.26	32.95	27.94	29.41
5NYS	RMSD (Å)	12.02	9.82	nc*	11.56
	INF	0.08	0.08	nc*	0.05
	GDT-TS (%)	31.25	37.50	nc*	33.75
5VHE	RMSD (Å)	13.11	15.95	nc*	16.46
	INF	0.00	0.00	nc*	0.24
	GDT-TS (%)	30.21	46.16	nc*	33.33
6EVV	RMSD (Å)	11.48	7.10	9.65	12.98
	INF	0.39	0.39	0.32	0.36
	GDT-TS (%)	44.23	52.78	45.19	43.27
6H1K	RMSD (Å)	11.86	10.45	14.16	16.63
	INF	0.45	0.45	0.46	0.47
	GDT-TS (%)	41.07	43.48	33.93	29.46
6SUU	RMSD (Å)	23.18	14.13	nc*	16.08
	INF	0.20	0.20	nc*	0.26
	GDT-TS (%)	24.22	27.68	nc*	24.22
6T2G	RMSD (Å)	26.09	13.50	nc*	16.06
	INF	0.07	0.07	nc*	0.13
	GDT-TS (%)	26.56	28.57	nc*	27.34
6ZL2	RMSD (Å)	17.55	13.48	20.57	10.91
	INF	0.49	0.49	0.57	0.40
	GDT-TS (%)	27.08	32.29	39.58	42.36
6ZL9	RMSD (Å)	15.76	10.92	20.30	39.80
	INF	0.61	0.61	0.69	0.67
	GDT-TS (%)	31.43	36.36	40.00	44.29

Continue on next page

Table S3- *Continued from previous page*

PDB	Metrics	RNAComposer	SimRNA	Vfold3D	3dDNA
6ZTE	RMSD (Å)	17.15	16.07	16.69	20.38
	INF	0.58	0.58	0.60	0.56
	GDT-TS (%)	34.02	32.29	39.58	42.36
7CLS	RMSD (Å)	13.90	12.09	15.25	16.97
	INF	0.32	0.32	0.52	0.32
	GDT-TS (%)	31.82	32.61	35.60	33.33
7CV3	RMSD (Å)	9.70	9.29	15.62	13.54
	INF	0.33	0.33	0.30	0.22
	GDT-TS (%)	41.67	53.12	35.19	37.04
7CV4	RMSD (Å)	7.99	8.11	11.55	14.01
	INF	0.32	0.32	0.34	0.37
	GDT-TS (%)	41.34	52.18	40.38	39.42
8AYG	RMSD (Å)	18.53	15.23	nc*	24.26
	INF	0.15	0.06	nc*	0.00
	GDT-TS (%)	25.81	24.19	nc*	31.45

nc: not calculated – these structures were not included in the predictions generated by the algorithm.*

Table S4: Summary of the structures not predicted by Vfold3D and 3dDNA. Secondary structures are represented using dot-bracket notation, where base pairs are denoted by matching opening and closing brackets, unpaired nucleotides by dots, and higher-order interactions with matching opening and closing square-brackets or braces. Following the Eltetrado notation, guanines involved in G-quadruplexes are also noted with sequential brackets and square-brackets.

**Tool**	**PDB**	**Method**	**length**	**G-quadruplex**	**Pseudoknot**	**Complex**	**i-motif**	**Experimental 2D**	**Predicted 2D**
Vfold3D	1OZ8	NMR	24	Yes	No	No	No	(..)..(..)...(..)..(..).	
	2KF8	NMR	22	Yes	No	No	No	((....)[.....)(...)]..	
	2N21	NMR	18	Yes	No	Yes	No	..([.)].([.)].	
	5NYS	NMR	20	Yes	No	No	No	..([..)].([..)].	
	5VHE	X-RAY	24	Yes	No	Yes	No	.([.)].([.)]........	
	6SUU	NMR	32	Yes	No	No	No	.([.)]...([...........)]....	
	6T2G	NMR	32	Yes	No	No	No	.([.)]..([............)]....	
	2A0I	X-RAY	10	No	No	Yes	No	....(..)..	
	5F55	X-RAY	14	No	No	Yes	No	......(....)..	
	5GWL	NMR	8	No	No	No	No	(..)(..)	
	5GWQ	NMR	8	No	No	No	No	(..)(..)	
	5OND	X-RAY	9	No	No	Yes	No	((....).)	
	6J37	NMR	8	No	No	No	No	(..)(..)	
	6M0B	NMR	8	No	No	No	No	(..)(..)	.((..)).
	6M0C	NMR	8	No	No	No	No	(..)(..)	
	8AYG	X-RAY	31	No	No	No	Yes	...((((...[[[[...))))...]]]]...	
3dDNA	1KR8	NMR	7	No	No	No	No	((...))	
	1PQT	NMR	7	No	No	No	No	((...))	

Table S5: Pairwise Wilcoxon signed-rank test results for RMSD, INF and GDT_TS metrics on the whole dataset including G4 and i-motif. Significance levels are indicated as follows: * $p < 0.05$, ** $p < 0.01$, *** $p < 0.001$.

Metric	Comparison	p-value	Significance
RMSD	RNAc vs SimRNA	0.092	—
	RNAc vs Vfold3D	0.407	—
	RNAc vs 3dDNA	1.273×10^{-6}	***
	SimRNA vs Vfold3D	0.914	—
	SimRNA vs 3dDNA	0.002	**
	Vfold3D vs 3dDNA	7.163×10^{-6}	***
INF	RNAc vs SimRNA	0.025	*
	RNAc vs Vfold3D	0.063	—
	RNAc vs 3dDNA	1.510×10^{-7}	***
	SimRNA vs Vfold3D	0.007	**
	SimRNA vs 3dDNA	2.327×10^{-11}	***
	Vfold3D vs 3dDNA	9.615×10^{-5}	***
GDT_TS	RNAc vs SimRNA	5.955×10^{-6}	***
	RNAc vs Vfold3D	2.338×10^{-7}	***
	RNAc vs 3dDNA	2.591×10^{-14}	***
	SimRNA vs Vfold3D	0.987	—
	SimRNA vs 3dDNA	6.786×10^{-9}	***
	Vfold3D vs 3dDNA	1.003×10^{-9}	***

Table S6: Pairwise Wilcoxon signed-rank test results for RMSD, INF and GDT_TS metrics on the subset excluding G4 and i-motif structures. Significance levels are indicated as follows: * $p < 0.05$, ** $p < 0.01$, *** $p < 0.001$.

Metric	Comparison	p-value	Significance
RMSD	RNAc vs SimRNA	0.344	—
	RNAc vs Vfold3D	0.005	**
	RNAc vs 3dDNA	3.652×10^{-11}	***
	SimRNA vs Vfold3D	0.002	**
	SimRNA vs 3dDNA	1.692×10^{-9}	***
	Vfold3D vs 3dDNA	3.738×10^{-8}	***
INF	RNAc vs SimRNA	0.032	*
	RNAc vs Vfold3D	0.407	—
	RNAc vs 3dDNA	1.552×10^{-7}	***
	SimRNA vs Vfold3D	0.092	—
	SimRNA vs 3dDNA	2.190×10^{-11}	***
	Vfold3D vs 3dDNA	3.419×10^{-7}	***
GDT_TS	RNAc vs SimRNA	0.007	**
	RNAc vs Vfold3D	7.357×10^{-7}	***
	RNAc vs 3dDNA	2.299×10^{-12}	***
	SimRNA vs Vfold3D	0.0396	*
	SimRNA vs 3dDNA	5.107×10^{-11}	***
	Vfold3D vs 3dDNA	2.068×10^{-9}	***

Table S7: Mean and median heavy atoms RMSD, total INF score and GDT_TS obtained for the RNAComposer, SimRNA, Vfold3D and 3dDNA on ssDNA models, including and excluding G4-containing structures.

**Methods**	**Metrics**		**G-quadruplex**	**not-G-quadruplex**	**total**
RNAComposer	RMSD (Å)	mean	13.64	6.45	8.38
		median	12.20	5.70	7.38
	INF	mean	0.30	0.69	0.59
		median	0.32	0.72	0.65
	GDT-TS (%)	mean	35.28	56.00	50.45
		median	33.93	53.90	48.44
SimRNA	RMSD (Å)	mean	10.87	6.82	7.91
		median	10.68	5.76	6.77
	INF	mean	0.30	0.62	0.53
		median	0.32	0.69	0.61
	GDT-TS (%)	mean	42.80	58.73	54.46
		median	43.04	57.50	53.12
Vfold3D	RMSD (Å)	mean	14.08	5.95	7.86
		median	14.15	5.38	6.62
	INF	mean	0.42	0.70	0.65
		median	0.46	0.73	0.69
	GDT-TS (%)	mean	39.26	60.49	55.51
		median	39.58	58.78	54.35
3dDNA	RMSD (Å)	mean	14.72	3.34	6.45
		median	13.57	1.90	3.23
	INF	mean	0.31	0.79	0.68
		median	0.34	0.90	0.84
	GDT-TS (%)	mean	37.91	83.23	70.83
		median	35.56	92.05	77.50

Table S8: Summary of the structures predicted by the whole Vfold algorithm

PDB	Method	length	G-quadruplex	Pseudoknot	Complex	i-motif
2KF8	NMR	22	Yes	No	No	No
2N21	NMR	18	Yes	No	Yes	No
5NYS	NMR	20	Yes	No	No	No
5VHE	X-RAY	24	Yes	No	Yes	No
6SUU	NMR	32	Yes	No	No	No
6T2G	NMR	32	Yes	No	No	No
2A0I	X-RAY	10	No	No	Yes	No
5F55	X-RAY	14	No	No	Yes	No
5GWL	NMR	8	No	No	No	No
5GWQ	NMR	8	No	No	No	No
5OND	X-RAY	9	No	No	Yes	No
6J37	NMR	8	No	No	No	No
6M0B	NMR	8	No	No	No	No
6M0C	NMR	8	No	No	No	No
8AYG	X-RAY	31	No	No	No	Yes

Table S9: GDT-TS, RMSD, and INF scores for the 14 predicted structures by the whole Vfold algorithm

Ref	GDT-TS (%)	RMSD (Å)	INF
2A0I	70.00	5.88	0.00
2KF8	34.09	13.07	0.04
2N21	37.50	11.31	0.43
5F55	41.07	7.17	0.47
5GWL	68.75	5.04	0.20
5GWQ	56.25	5.30	0.21
5NYS	38.75	13.21	0.41
5OND	66.67	4.68	0.58
5VHE	29.17	18.95	0.38
6J37	68.75	4.84	0.18
6M0B	68.75	4.97	0.18
6M0C	68.75	5.22	0.26
6SUU	25.78	18.40	0.37
6T2G	25.78	23.29	0.25
8AYG			

Table S10: Comparison of 1SNJ suboptimal structures predicted by RNAComposer, Vfold3D, SimRNA and 3dDNA based on RMSD, INF and GDT scores.

	Metrics	RNAComposer	Vfold3D	SimRNA	3dDNA
Model1	RMSD (Å)	9.57	8.32	8.83	3.45
	INF	0.66	0.67	0.66	0.89
	GDT-TS (%)	34.72	36.11	38.19	93.06
Model2	RMSD	9.86	6.36	7.51	4.46
	INF	0.77	0.65	0.65	0.87
	GDT-TS (%)	38.88	34.72	40.97	85.42
Model3	RMSD	10.40	7.45	7.91	4.80
	INF	0.64	0.55	0.66	0.79
	GDT-TS (%)	31.25	38.89	38.89	81.25
Model4	RMSD	11.12	5.85	10.32	4.18
	INF	0.62	0.64	0.66	0.83
	GDT-TS (%)	35.42	36.80	32.64	86.80
Model5	RMSD	9.94	9.74	10.63	9.76
	INF	0.67	0.64	0.62	0.71
	GDT-TS (%)	35.42	31.25	38.20	42.36
Model6	RMSD	9.96			
	INF	0.65			
	GDT-TS (%)	41.67			
Model7	RMSD	10.33			
	INF	0.62			
	GDT-TS (%)	31.25			
Model8	RMSD	11.09			
	INF	0.67			
	GDT-TS (%)	30.55			
Model9	RMSD	9.46			
	INF	0.67			
	GDT-TS (%)	36.80			
Model10	RMSD	8.99			
	INF	0.71			
	GDT-TS (%)	38.89			

Table S11: Median heavy atoms RMSD, total INF score, and GDT_TS obtained for the RNAcomposer, SimRNA Vfold3D, and 3dDNA models in complex with a protein, in the free state, or both, including, excluding or focusing on G4-containing structures.

**Method**	**Metric**	**Complex**		**Free**		**All dataset**	**All - (G4 & i-motif)**	**Only G4**
		G4 incl.	G4 excl.	G4 incl.	G4 excl.			
RNAcomposer	RMSD	7.29	6.48	7.57	4.33	7.38	5.70	12.21
	INF	0.71	0.75	0.51	0.70	0.65	0.72	0.32
	GDT_TS	48.75	51.00	45.04	62.50	48.44	54.12	33.93
SimRNA	RMSD	6.77	6.38	6.90	4.44	6.77	5.74	10.68
	INF	0.68	0.70	0.50	0.67	0.60	0.69	0.32
	GDT_TS	53.57	54.41	52.65	62.50	53.57	58.54	43.04
Vfold3D	RMSD	6.22	5.63	7.19	4.24	6.62	5.38	14.16
	INF	0.72	0.74	0.66	0.73	0.69	0.74	0.46
	GDT_TS	55.71	57.42	47.50	66.03	54.35	58.78	39.58
3dDNA	RMSD	3.23	2.11	3.03	1.23	3.23	1.89	13.57
	INF	0.87	0.90	0.67	0.89	0.84	0.90	0.34
	GDT_TS	83.34	87.50	76.14	95.83	77.50	92.08	35.56

Table S12: Comparison of suboptimal structures predicted for 4HT4 by RNAComposer, Vfold3D, SimRNA and 3dDNA based on RMSD, INF and GDT-TS scores.

	**Metrics**	**RNAComposer**	**Vfold3D**	**SimRNA**	**3dDNA**
Model1	RMSD (Å)	11.93	12.48	16.47	9.64
	INF	0.65	0.68	0.65	0.74
	GDT	44.64	43.75	39.29	60.71
Model2	RMSD	12.44	11.77		9.42
	INF	0.58	0.71		0.82
	GDT	36.61	45.54		62.50
Model3	RMSD	12.83	12.81		9.54
	INF	0.65	0.69		0.71
	GDT	38.39	41.97		62.50
Model4	RMSD	12.18	12.41		9.53
	INF	0.65	0.63		0.72
	GDT	39.29	42.86		65.18
Model5	RMSD	11.64	12.93		9.49
	INF	0.60	0.65		0.76
	GDT	40.18	42.86		61.61
Model6	RMSD	12.77			
	INF	0.65			
	GDT	38.39			
Model7	RMSD	12.53			
	INF	0.65			
	GDT	37.50			
Model8	RMSD	11.74			
	INF	0.64			
	GDT	38.39			
Model9	RMSD	11.02			
	INF	0.60			
	GDT	39.29			
Model10	RMSD	12.62			
	INF	0.64			
	GDT	41.07			

Table S13: Heavy atoms RMSD values (Å) of suboptimal structures containing G4 motif generated by the four tools RNAComposer, SimRNA, Vfold3D and 3dDNA

Method	Refs	model1	model2	model3	model4	model5	model6	model7	model8	model9	model10
**RNAComposer**	148D	9.42	9.71	9.41	9.63	9.50	9.79	9.89	9.50	10.42	9.26
	1HAO	9.11	13.86	7.90	16.53	19.73	11.25	15.29	11.45	12.38	13.00
	1I34	11.31	12.82	11.16	10.25	12.81	11.97	12.21	12.55	10.80	10.76
	1OZ8	18.79	13.93	17.27	17.09	15.33	19.35	13.79	18.17	16.95	19.36
	2HY9	20.00	17.44	16.02	15.47	19.59	15.71	19.71	16.85	27.43	19.71
	2KF8	12.43	13.23	13.61	15.50	13.85	20.77	12.70	14.38	14.63	15.08
	2M8Z	9.88	9.02	9.39	7.70	8.84	7.98	8.00	6.80	10.98	9.75
	2M90	10.53	18.43	21.98	10.87	11.85	10.78	11.50	12.13	14.54	13.83
	2M91	10.38	10.11	10.16	11.36	9.73	10.23	11.66	10.76	11.02	9.35
	2M92	11.66	15.32	14.42	17.62	14.46	14.60	13.32	15.93	16.81	12.97
	2M93	12.39	11.84	11.84	13.71	13.44	12.54	13.34	12.11	12.15	13.02
	2N21	14.14	14.11	14.32	14.66	10.53	14.54	9.99	11.83	12.17	12.21
	5CMX	9.66	9.77	9.15	9.69	9.10	10.28	9.67	9.43	9.79	9.40
	5MTA	18.79	17.29	19.69	18.15	17.15	19.05	16.60	21.23	19.07	16.55
	5NYS	12.02	15.36	11.49	11.79	11.59	12.94	12.29	14.98	11.63	13.05
	5VHE	13.11	12.19	12.29	12.30	12.21	14.04	14.01	12.67	11.78	12.62
	6EVV	11.48	12.27	11.80	11.90	12.03	11.91	11.97	11.73	12.17	11.67
	6H1K	11.86	16.22	16.25	13.80	13.07	13.48	12.18	13.57	11.42	11.35
	6SUU	23.18	17.31	20.02	19.55	14.56	16.16	17.60	15.76	18.38	17.49
	6T2G	34.77	18.92	17.66	26.10	27.43	15.99	20.52	19.80	21.52	21.99
	6ZL2	17.55	19.46	16.79	17.49	17.35	18.14	18.10	19.90	19.61	17.37
	6ZL9	15.76	14.60	17.06	13.94	15.10	13.65	16.56	16.86	15.49	15.54
	6ZTE	17.15	13.93	11.84	15.09	12.73	12.76	12.29	16.13	16.03	13.67
	7CLS	13.90	14.81	14.38	13.70	13.33	14.01	14.35	13.33	14.34	13.29
	7CV3	9.70	12.26	14.13	11.69	12.87	12.15	11.14	12.41	15.39	12.08
	7CV4	7.99	8.34	9.15	11.64	7.93	8.48	9.00	10.02	8.75	8.86
**SimRNA**	148D	7.17	7.16	7.07	6.29	8.61	5.55	7.18	6.33	9.27	9.94
	1HAO	7.44	6.79	6.78	6.79	7.38	7.42	6.61	6.81	7.40	6.86
	1I34	9.88	9.92	9.97	10.28	9.86	9.83	10.09	10.29	9.94	9.99
	1OZ8	10.00	17.75	18.77	14.53	12.25	16.93	17.45	16.25	18.94	17.26
	2HY9	11.35	11.65	11.22	12.57	11.19	11.69	11.60	12.59	11.48	11.91
	2KF8	12.17	11.04	11.01	10.19	10.67	11.08	12.90	13.05	10.36	10.82
	2M8Z	6.45	7.02	6.53	6.92	6.25	5.72	8.22	7.60	6.63	7.01
	2M90	7.77	14.48	14.60	13.74	13.00	13.60	12.90	6.48	12.65	13.52
	2M91	9.52	9.44	8.77	9.00	8.93	8.95	8.79	9.56	9.23	8.36
	2M92	11.59	11.75	12.02	12.24	11.58	11.93	11.45	11.48	13.71	13.43
	2M93	10.24	11.27	10.22	10.46	10.56	11.44	10.11	10.79	10.03	10.56
	2N21	10.43	9.31	10.32	10.53	10.01	9.95	10.28	10.55	9.76	9.24
	5CMX	6.76	9.45	7.57	8.95	9.79	9.03	9.49	9.52	9.11	7.54
	5MTA	13.28	11.13	10.97	10.92	11.44	13.69	14.81	13.49	12.49	11.19
	5NYS	9.75	10.11	10.28	9.57	10.29	10.30	10.30	10.21	10.30	10.71
	5VHE	17.32	17.38	16.90	16.68	19.67	17.26	17.10	17.81	17.38	17.02
	6EVV	7.63	8.54	8.25	7.64	7.50	7.96	8.37	7.43	7.45	8.14
	6H1K	10.12	10.70	10.19	9.73	8.32	10.71	10.46	10.93	10.15	11.23
	6SUU	13.76	13.84	14.11	14.36	14.09	14.01	13.75	13.87	14.52	13.98
	6T2G	13.35	14.61	13.33	14.60	13.77	13.66	12.93	14.89	13.10	14.43
	6ZL2	16.39	10.76	14.36	12.21	9.77	12.66	12.42	14.84	12.60	16.36
	6ZL9	10.50	11.13	10.99	11.16	14.24	11.53	12.34	12.46	11.79	13.43
	6ZTE	16.69	11.32	11.97	10.54	17.17	11.05	11.06	15.82	16.95	15.62
	7CLS	11.84	12.79	11.59	11.98	nan	11.92	12.53	11.53	12.32	11.39
	7CV3	9.19	11.16	7.72	8.05	8.18	9.36	9.75	8.25	7.67	9.95
	7CV4	9.25	9.55	8.14	9.20	9.75	8.39	8.64	9.69	8.21	9.57
**Vfold3D**	148D	9.26	8.32	7.77	7.75	7.66	–	–	–	–	–
	1HAO	11.80	12.07	12.43	11.14	11.87	–	–	–	–	–
	1I34	16.61	15.74	18.22	15.17	15.37	–	–	–	–	–
	2HY9	23.54	17.25	25.00	26.79	25.81	–	–	–	–	–
	2M8Z	5.99	6.72	8.01	5.44	9.78	–	–	–	–	–
	2M90	14.95	15.39	16.18	15.04	15.31	–	–	–	–	–

Continue on next page

Table S13- *Continued from previous page*

Method	Refs	model1	model2	model3	model4	model5	model6	model7	model8	model9	model10
	2M91	9.02	9.68	13.40	7.86	11.16	–	–	–	–	–
	2M92	13.38	13.30	11.86	12.33	13.61	–	–	–	–	–
	2M93	12.86	13.09	13.20	13.96	12.78	–	–	–	–	–
	5CMX	7.94	8.30	9.82	9.27	7.82	–	–	–	–	–
	5MTA	18.31	18.91	21.37	14.05	14.59	–	–	–	–	–
	6EVV	9.65	7.59	13.18	11.18	11.33	–	–	–	–	–
	6H1K	14.16	17.00	20.00	18.49	14.62	–	–	–	–	–
	6ZL2	20.58	18.52	19.03	15.04	18.18	–	–	–	–	–
	6ZL9	20.30	18.30	18.70	16.11	19.24	–	–	–	–	–
	6ZTE	16.69	17.29	19.28	20.99	18.60	–	–	–	–	–
	7CLS	15.25	15.26	17.55	13.82	16.67	–	–	–	–	–
	7CV3	15.62	13.11	16.34	15.61	16.09	–	–	–	–	–
	7CV4	11.55	10.40	11.60	10.90	10.26	–	–	–	–	–
**3dDNA**	148D	14.25	14.25	11.84	14.25	11.81	–	–	–	–	–
	1HAO	13.60	13.60	11.28	13.60	11.25	–	–	–	–	–
	1I34	8.74	8.74	11.59	8.74	11.59	–	–	–	–	–
	1OZ8	11.02	13.43	13.97	21.55	16.64	–	–	–	–	–
	2HY9	13.47	20.16	17.41	12.14	16.76	–	–	–	–	–
	2KF8	16.00	16.00	16.52	16.00	16.52	–	–	–	–	–
	2M8Z	7.46	6.30	7.92	7.23	8.07	–	–	–	–	–
	2M90	12.40	12.01	10.00	15.42	12.58	–	–	–	–	–
	2M91	10.51	10.56	10.71	10.69	10.48	–	–	–	–	–
	2M92	13.39	11.42	13.62	11.39	10.41	–	–	–	–	–
	2M93	10.35	10.42	10.39	10.43	10.41	–	–	–	–	–
	2N21	14.99	15.65	12.98	18.62	11.98	–	–	–	–	–
	5CMX	12.00	12.13	15.22	15.18	17.15	–	–	–	–	–
	5MTA	19.08	13.99	16.70	11.86	14.71	–	–	–	–	–
	5NYS	11.56	11.56	10.86	11.56	10.86	–	–	–	–	–
	5VHE	16.46	12.81	17.88	14.04	13.63	–	–	–	–	–
	6EVV	12.98	12.98	16.21	19.42	17.03	–	–	–	–	–
	6H1K	16.63	16.45	16.33	16.61	16.96	–	–	–	–	–
	6SUU	16.08	16.08	16.08	16.08	16.08	–	–	–	–	–
	6T2G	16.06	16.06	16.06	16.06	16.06	–	–	–	–	–
	6ZL2	10.91	14.06	17.30	14.81	12.25	–	–	–	–	–
	6ZL9	39.80	39.99	40.06	39.92	39.89	–	–	–	–	–
	6ZTE	20.38	20.32	20.35	20.43	20.41	–	–	–	–	–
	7CLS	16.97	11.37	15.12	12.39	14.81	–	–	–	–	–
	7CV3	13.54	8.09	11.66	14.72	11.02	–	–	–	–	–
	7CV4	14.01	12.38	11.34	16.55	13.26	–	–	–	–	–

Table S14: AptaMat score computed between the experimental and the models secondary structures. When the AptaMat score could not be computed (absence of secondary structure in one of the two compared dot-bracket sequences, a value of -1 has been indicated.

Refs	RNAComposer	SimRNa	Vfold3D	3dDNA
148D	-1.00	4.00	-1.00	-1.00
1AC7	0.00	0.18	0.00	0.00
1B4Y	0.18	0.15	0.00	0.73
1BJH	0.00	0.29	0.00	0.00
1D16	0.00	0.00	0.00	0.00
1ECU	0.00	0.00	0.00	0.00
1EN1	0.00	0.67	0.00	0.43
1EZN	0.00	0.00	0.00	0.00
1HAO	-1.00	4.25	-1.00	-1.00
1I34	11.40	6.71	-1.00	6.00
1JVE	0.00	0.29	0.00	0.00
1KR8	0.00	0.00	0.00	-1.00
1LA8	0.00	0.00	0.00	0.00
1NGO	0.00	0.00	0.10	0.00
1NGU	0.00	0.00	0.00	0.00
1OSB	0.00	2.38	0.50	0.00
1OZ8	-1.00	-1.00	-1.00	-1.00
1P0U	0.00	0.00	0.00	0.00
1PQT	0.00	0.00	0.00	-1.00
1SNJ	0.00	0.00	0.00	0.00
1UUT	0.00	0.00	0.00	0.00
1XUE	0.00	0.50	4.00	0.00
1YTB	0.00	0.00	0.00	0.00
1ZHU	0.00	0.60	0.00	0.00
1ZM5	0.00	0.50	0.18	0.00
2A0I	0.00	5.00	-1.00	0.00
2A6O	0.00	0.00	0.00	0.00
2EXF	0.00	0.43	0.00	0.00
2F1Q	0.00	0.00	0.11	0.00
2HY9	-1.00	10.50	-1.00	-1.00
2JZW	0.00	0.43	0.00	0.00
2K71	0.00	0.00	0.00	0.00
2KF8	-1.00	7.25	0.00	-1.00
2L5K	0.00	0.20	0.00	0.00
2LO5	0.00	0.00	0.60	0.00
2LO8	0.00	0.00	0.00	0.00
2M8Y	0.00	0.00	3.50	0.00
2M8Z	3.50	2.63	8.89	2.82
2M90	8.89	8.89	4.12	8.89
2M91	4.12	4.12	9.68	4.12
2M92	1.76	1.45	7.88	11.06
2M93	8.25	8.81	0.00	7.88
2N21	-1.00	2.78	-1.00	-1.00
2N8A	0.00	0.00	0.00	0.00
2VHG	0.00	0.25	0.00	0.00
2VIC	0.00	0.12	0.00	0.00
2VJU	0.10	0.36	0.00	0.00
3C46	0.00	2.77	0.18	0.00
3DSD	0.00	0.00	0.00	0.00
3HXO	0.00	0.32	0.00	0.13
3Q0A	0.00	1.83	0.00	0.00
3Q23	0.00	2.77	0.18	0.00
3Q24	0.00	2.77	0.00	0.00
3THW	0.00	0.00	0.00	0.04
3WPD	0.00	1.00	0.00	0.00
3WPG	0.00	1.00	-1.00	0.00
3WPH	0.00	0.00	0.00	0.00
3ZH2	0.00	3.31	0.38	0.00

Continue on next page

Table S14- *Continued from previous page*

Refs	RNAComposer	SimRNa	Vfold3D	3dDNA
4ER8	0.00	0.00	0.00	0.00
4F41	0.07	0.07	0.00	0.00
4F43	0.07	0.00	0.07	0.00
4FF1	0.00	2.00	0.00	0.00
4HT4	0.00	1.79	0.00	0.00
4I7Y	2.00	1.00	2.00	3.22
4KB0	0.00	0.17	0.33	0.00
4KB1	0.00	0.33	0.33	0.00
5CMX	2.25	1.50	2.00	2.75
5F55	0.00	2.00	-1.00	-1.00
5GWL	0.00	2.67	-1.00	0.00
5GWQ	0.00	-1.00	-1.00	0.00
5HRU	1.20	1.20	1.20	0.00
5HTO	1.09	0.00	1.09	0.00
5MTA	13.25	6.87	-1.00	16.50
5N2Q	0.00	0.00	0.00	0.00
5NYS	-1.00	7.86	-1.00	-1.00
5OND	0.00	0.50	-1.00	0.00
5VHE	6.86	9.08	-1.00	-1.00
6EVV	2.57	1.88	2.57	3.92
6FK4	0.00	0.00	0.00	0.00
6FK5	0.00	0.00	0.00	0.00
6FKE	0.00	0.00	0.00	0.00
6H1K	10.25	3.47	10.25	10.25
6IY5	0.00	0.00	-1.00	0.00
6J37	0.00	2.67	-1.00	0.00
6M0B	0.00	3.33	-1.00	0.00
6M0C	0.00	3.67	-1.00	0.00
6SEI	0.00	0.09	0.00	0.00
6SUU	-1.00	8.80	-1.00	-1.00
6T2G	-1.00	10.40	-1.00	-1.00
6U82	0.00	0.00	0.00	0.00
6ZL2	9.90	3.90	9.90	11.22
6ZL9	9.90	3.17	9.90	9.90
6ZTE	9.90	6.39	9.90	9.90
7CLS	8.50	1.71	8.50	8.50
7CV3	7.25	4.75	7.25	7.25
7CV4	3.50	4.00	6.00	6.00
8AYG	-1.00	8.67	-1.00	8.67

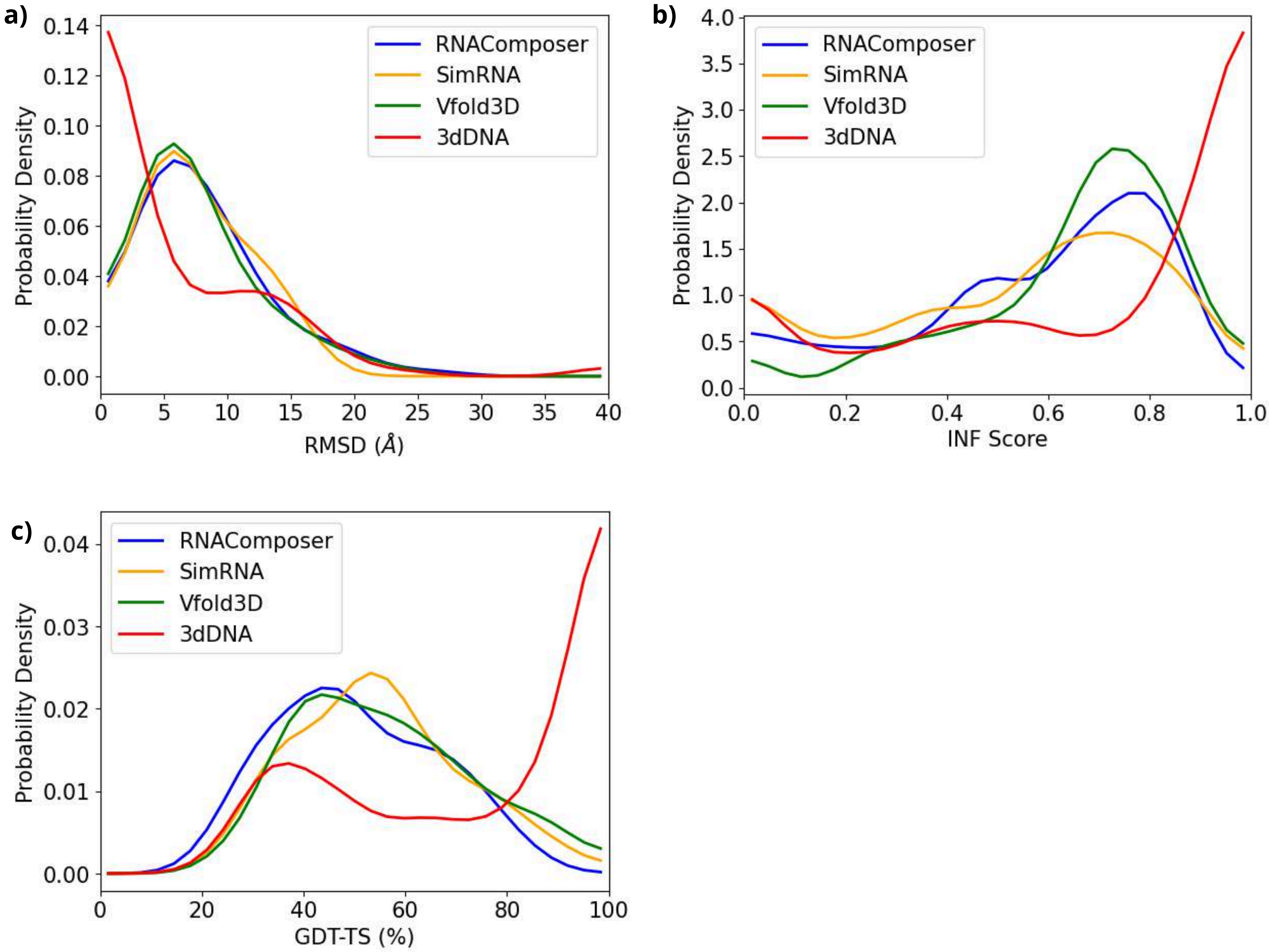

Figure S1: Distributions of (a) heavy atoms RMSD, (b) INF score, and (c) GDT-TS obtained for the models provided by RNAComposer (blue), SimRNA (yellow), Vfold3D (green) and 3dDNA (red), respectively.

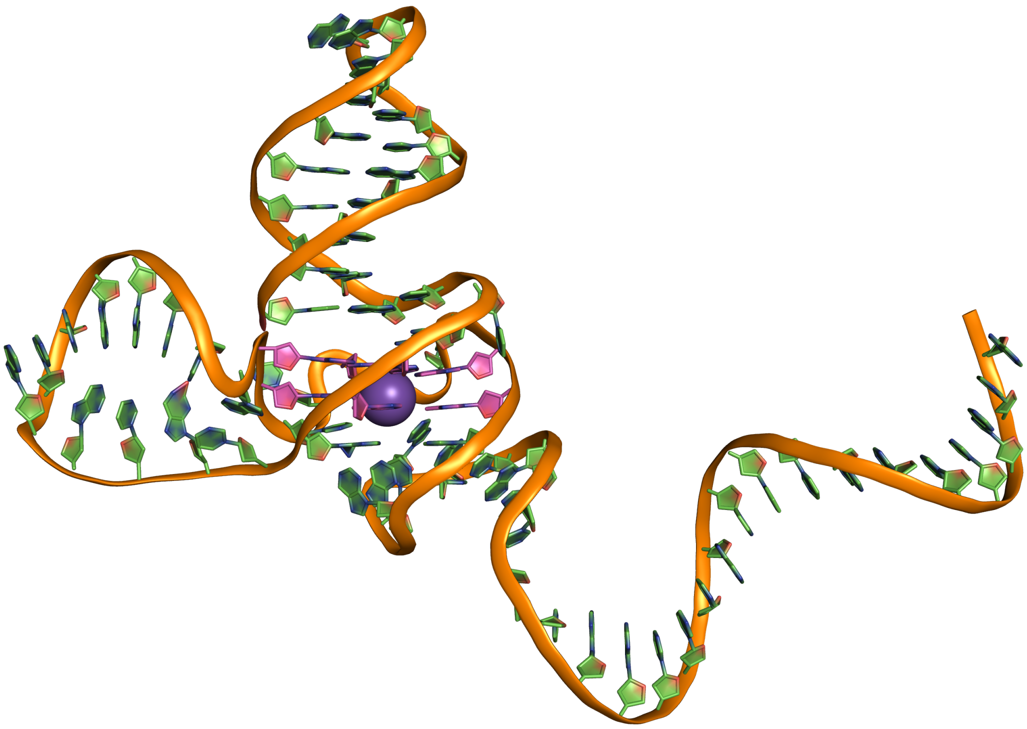

Figure S2: AlphaFold3-predicted 3D structure of an aptamer targeting Borrelia burgdorferi CspZ protein, showing the formation of a parallel G-quadruplex motif stabilized by K+ ions.

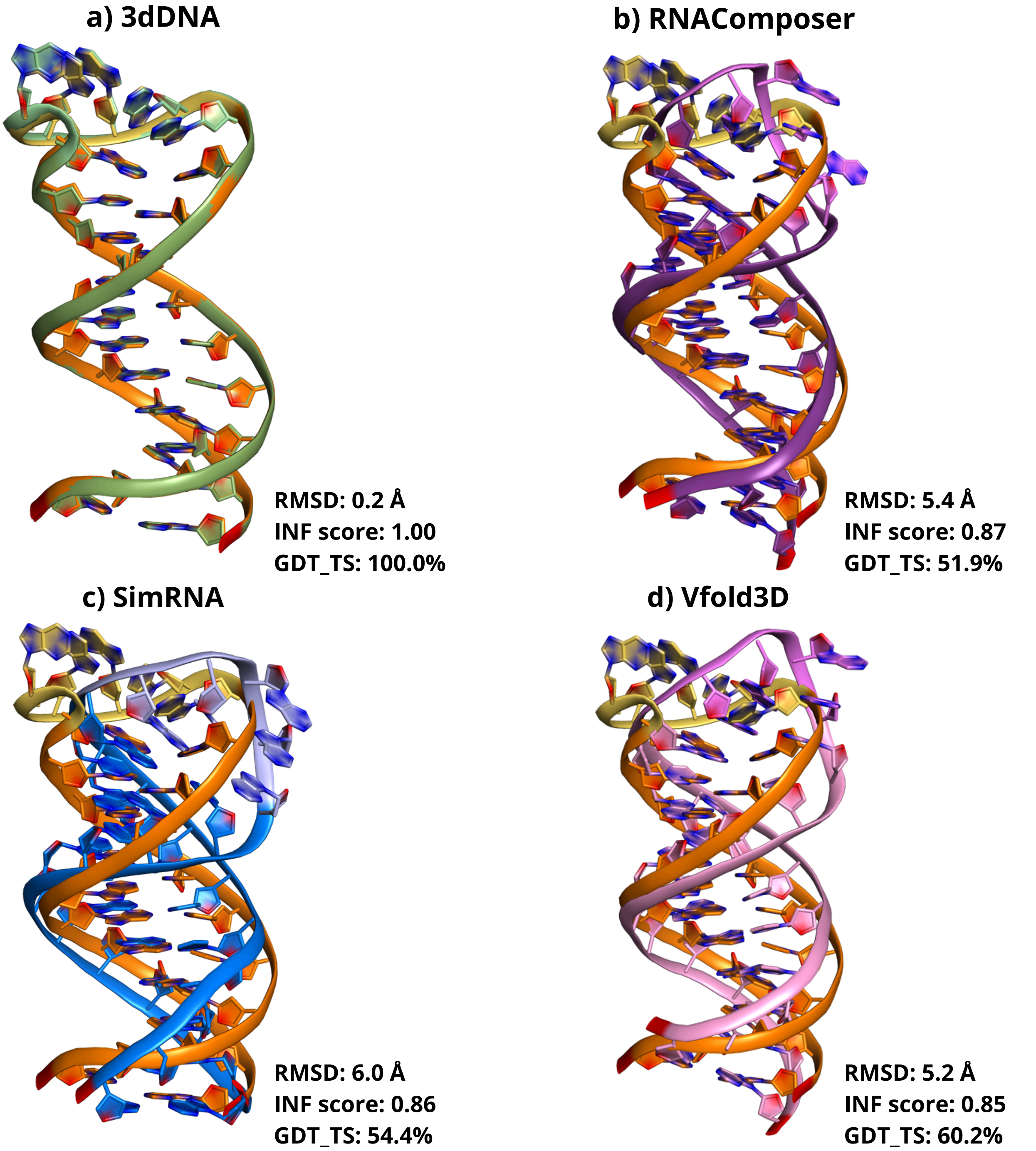

Figure S3: Alignment of the a) 3dDNA b) RNAComposer, c) SimRNA, and d) Vfold3D predicted structures to the experimental structure of the 1NGO ssDNA. The experimental structure hairpin stem-loop and long loop are colored in orange and yellow, respectively.

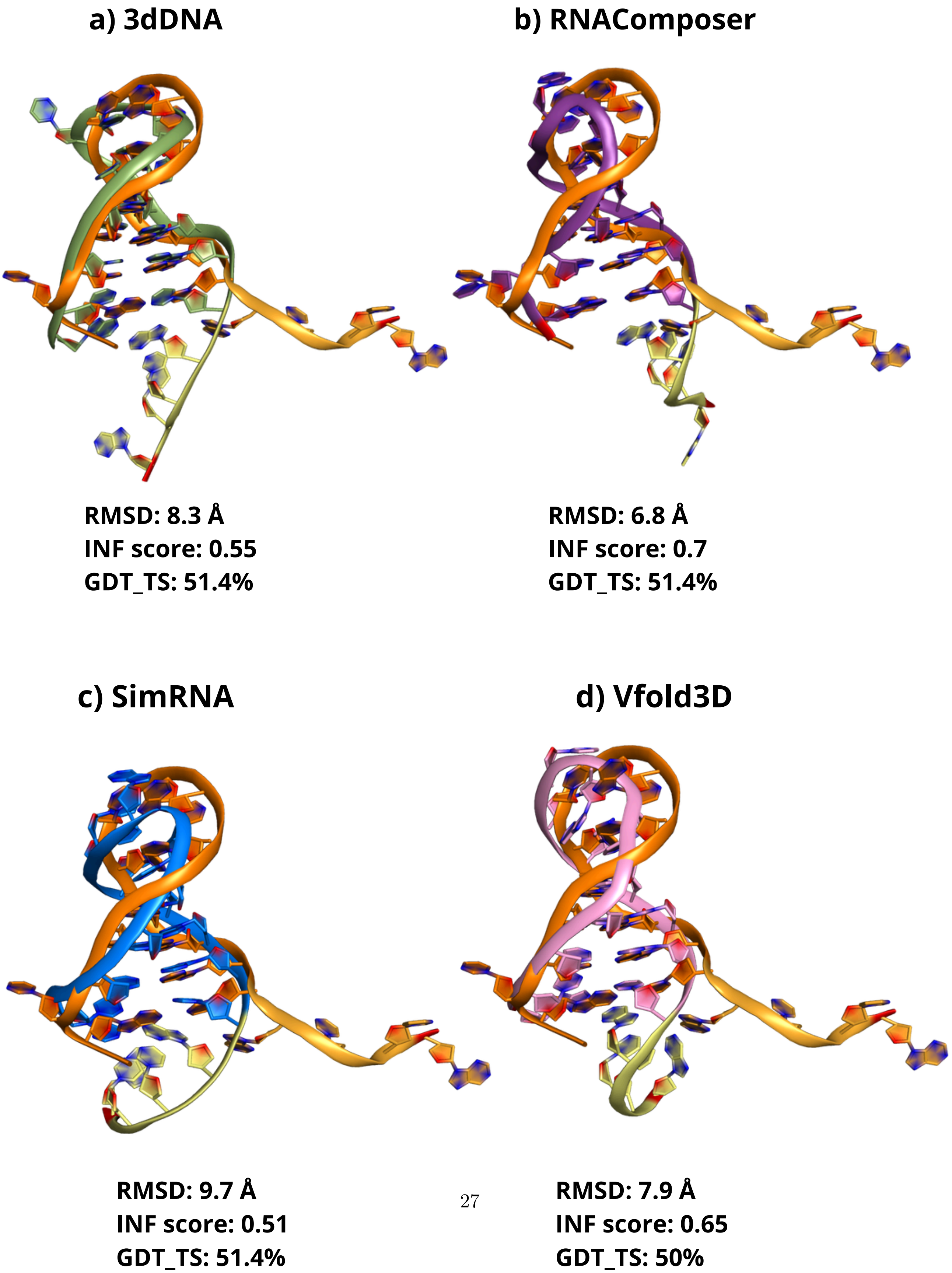

Figure S4: Alignment of the a) 3dDNA, b) RNAComposer, c) SimRNA, and d) Vfold3D predicted structures to the experimental structure of the 1EN1 ssDNA. The alignment has been realized by focusing on the only hairpin stem-loop motif phosphate backbone. The experimental structure hairpin stem-loop and unpaired extremity are colored in orange and yellow, respectively.

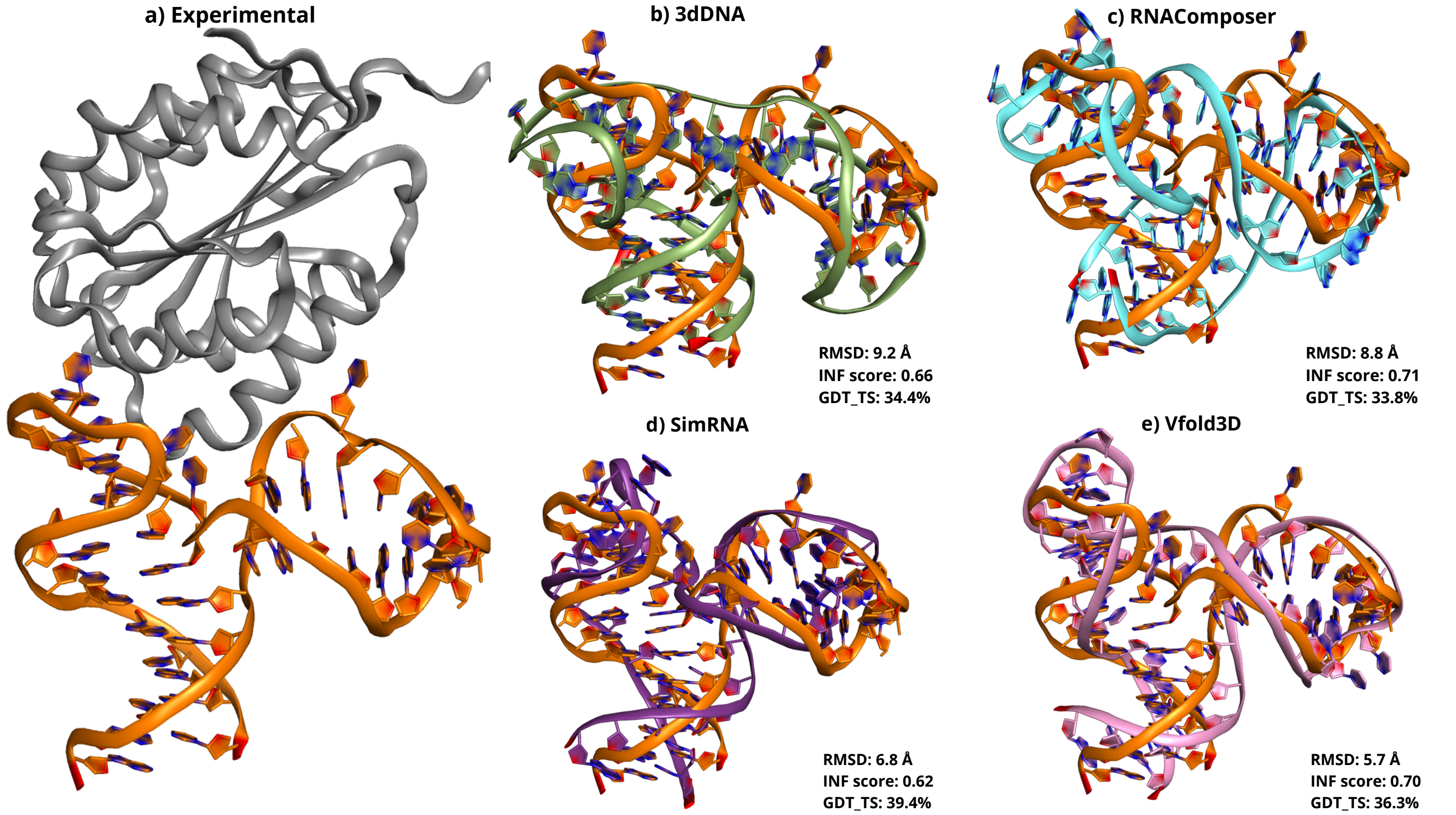

Figure S5: Alignment of the a) experimental b) 3dDNA c) RNAComposer, d) SimRNA, and e) Vfold3D predicted structures to the experimental structure of the 3HXO ssDNA.

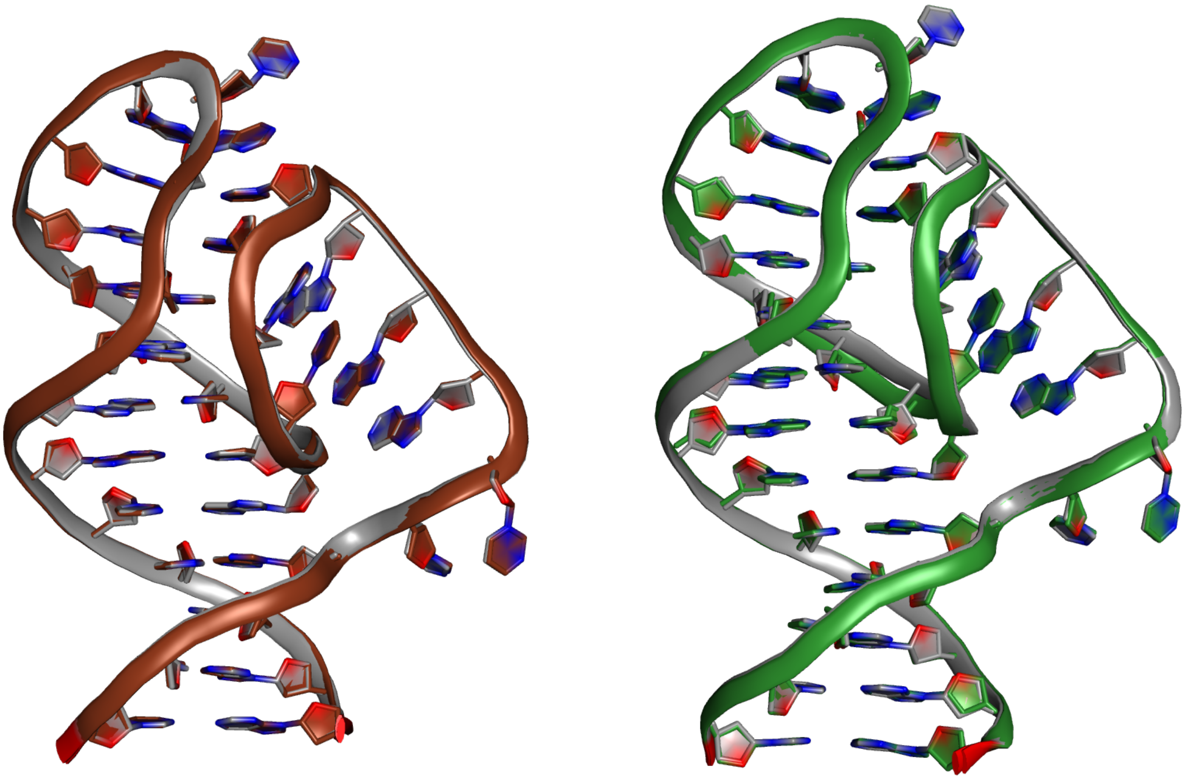

Figure S6: Alignment of the predicted 5HRU (left) and 5HTO (right) structures generated by 3dDNA without excluding their corresponding experimental structures.

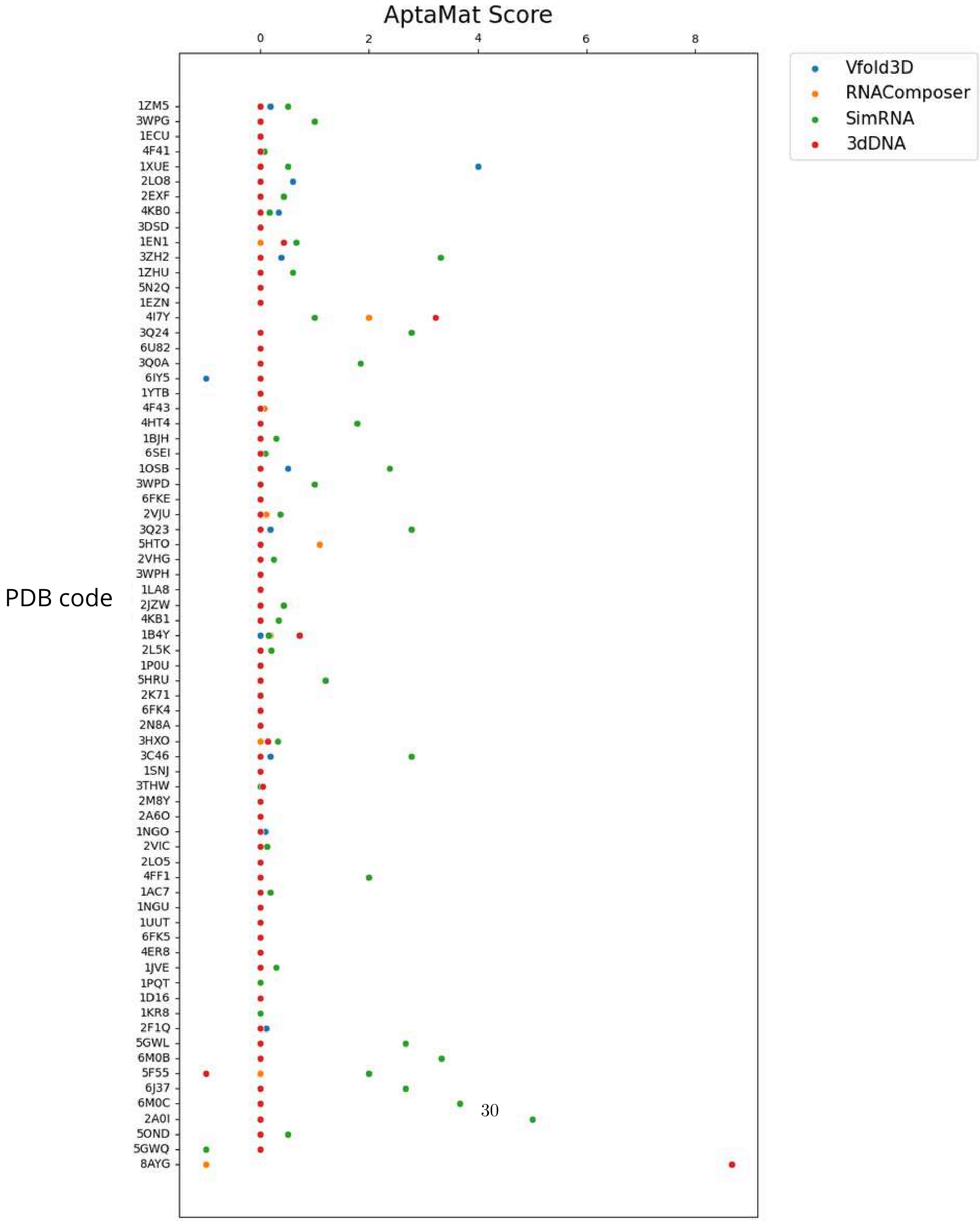

Figure S7: AptaMat scores for the oligonucleotides secondary structures of the models obtained using RNAComposer (orange), Vfold3D (blue), SimRNA (green) and 3dDNA (red). G4-containing models were excluded. None values are replaced by -1.

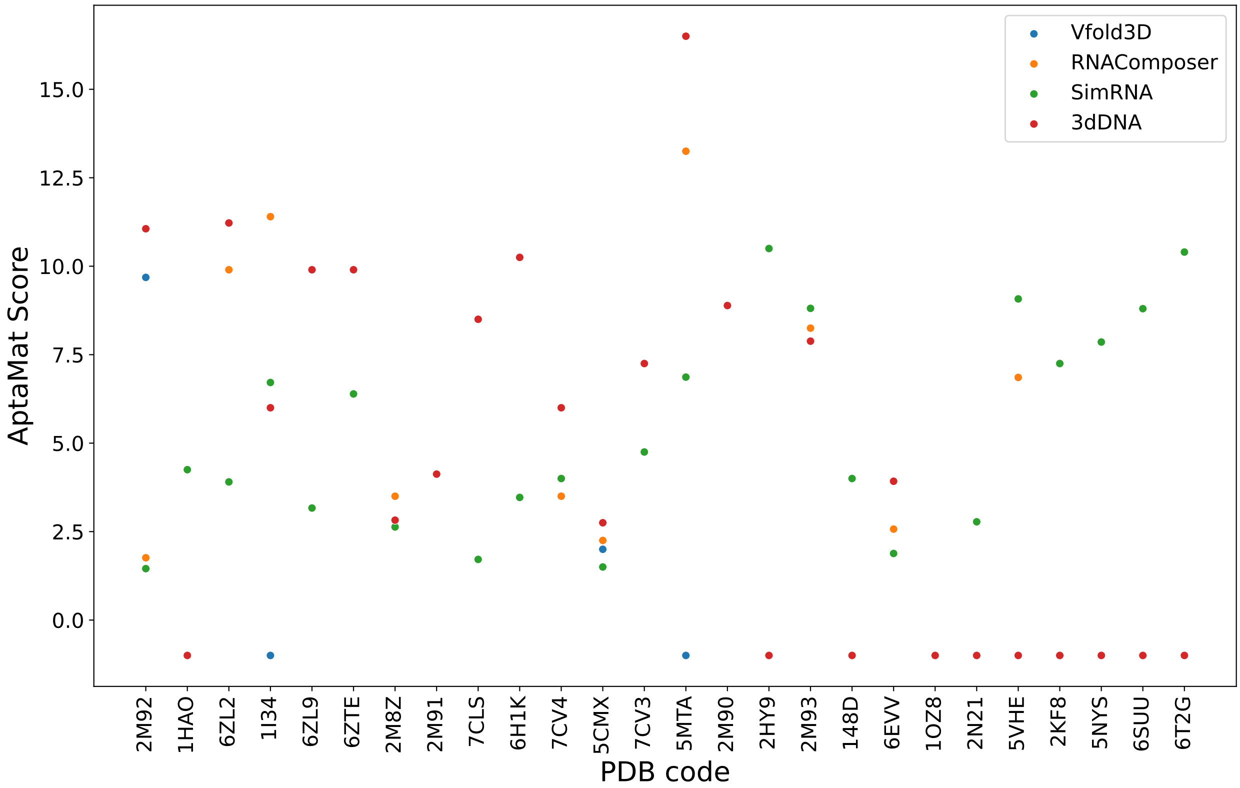

Figure S8: AptaMat scores for the oligonucleotides secondary structures of the G4-containing models obtained using RNA-Composer (orange), Vfold3D (blue), SimRNA (green) and 3dDNA (red). None values are replaced by -1.

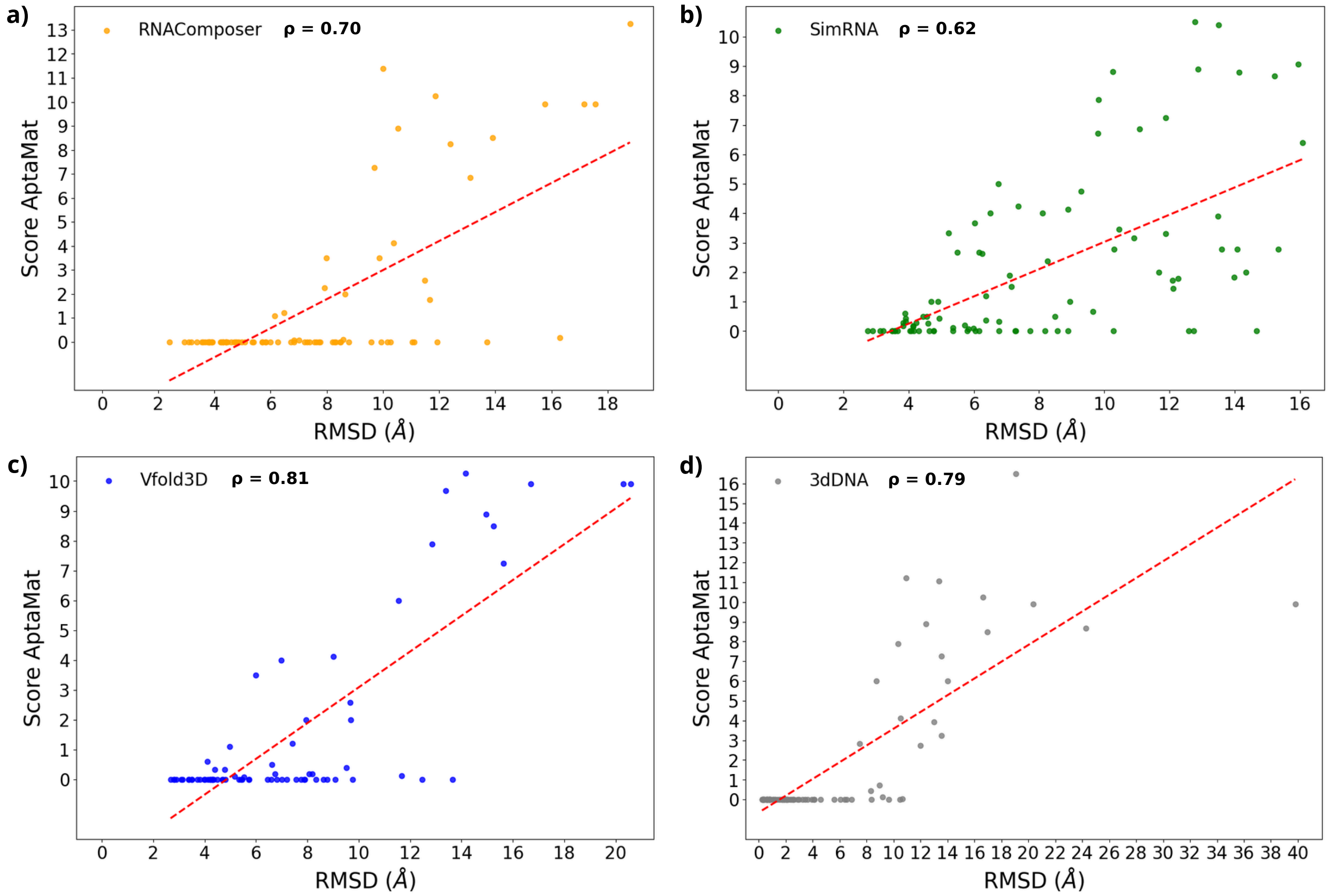

Figure S9: Correlation between AptaMat scores and RMSD values of the 3D structures predicted by RNAComposer, SimRNA, Vfold3D and 3dDNA.
